# Extensor carpi ulnaris muscle shows unexpected slow-to-fast fiber-type switch in Duchenne muscular dystrophy dogs

**DOI:** 10.1242/dmm.049006

**Published:** 2021-12-16

**Authors:** Chady H. Hakim, Hsiao T. Yang, Matthew J. Burke, James Teixeira, Gregory J. Jenkins, N. Nora Yang, Gang Yao, Dongsheng Duan

**Affiliations:** 1 Department of Molecular Microbiology and Immunology, School of Medicine, The University of Missouri, Columbia, MO 65212, USA; 2 National Center for Advancing Translational Sciences, National Institutes of Health, Bethesda, MD 20892, USA; 3 Department of Biomedical, Biological and Chemical Engineering, College of Engineering, The University of Missouri, Columbia, MO 65212, USA; 4 Department of Neurology, School of Medicine, The University of Missouri, Columbia, MO 65212, USA; 5 Department of Biomedical Sciences, College of Veterinary Medicine, The University of Missouri, Columbia, MO 65212, USA

**Keywords:** Dystrophin, Duchenne muscular dystrophy, Canine, Fiber type, New method, Contraction kinetics

## Abstract

Aged dystrophin-null canines are excellent models for studying experimental therapies for Duchenne muscular dystrophy, a lethal muscle disease caused by dystrophin deficiency. To establish the baseline, we studied the extensor carpi ulnaris (ECU) muscle in 15 terminal age (3-year-old) male affected dogs and 15 age/sex-matched normal dogs. Affected dogs showed histological and anatomical hallmarks of dystrophy, including muscle inflammation and fibrosis, myofiber size variation and centralized myonuclei, as well as a significant reduction of muscle weight, muscle-to-body weight ratio and muscle cross-sectional area. To rigorously characterize the contractile properties of the ECU muscle, we developed a novel *in situ* assay. Twitch and tetanic force, contraction and relaxation rate, and resistance to eccentric contraction-induced force loss were significantly decreased in affected dogs. Intriguingly, the time-to-peak tension and half-relaxation time were significantly shortened in affected dogs. Contractile kinetics predicted an unforeseen slow-to-fast myofiber-type switch, which we confirmed at the protein and transcript level. Our study establishes a foundation for studying long-term and late-stage therapeutic interventions in dystrophic canines. The unexpected myofiber-type switch highlights the complexity of muscle remodeling in dystrophic large mammals.

This article has an associated First Person interview with the first author of the paper.

## Abstract (precis)

**Summary:** A slow-to-fast fiber-type switch in dystrophic canine ECU muscle is revealed by contraction kinetics and myosin protein and transcript expression. This highlights the complexity of muscle remodeling in Duchenne muscular dystrophy.

## INTRODUCTION

Duchenne muscular dystrophy (DMD) is the most severe form of muscular dystrophy, affecting approximately one in every 5000 male births (Duan et al., 2021). DMD is caused by the loss of dystrophin, a subsarcolemmal structural protein critical for preserving sarcolemmal integrity and for assisting lateral and longitudinal force transmission during contraction (Hughes et al., 2015; Kunkel, 2005; Peter et al., 2011). Dystrophin-deficient muscle becomes sensitive to contraction-induced injury, undergoes degeneration and loses force production capacity. Eventually, patients are immobilized to a wheelchair in their early teens and die before 30 years of age (Duan et al., 2021). With improved cardiorespiratory support and care, patients can now live to their forties (Kieny et al., 2013).

There has been tremendous progress in translating experimental therapeutics from animal models to human patients in recent years (Verhaart and Aartsma-Rus, 2019). However, most of these studies are conducted on young animals and pre-teenage patients. Given the improvement in lifespan, there is an urgent need to evaluate (1) the safety and efficacy of newly developed therapies in subjects that are at the advanced disease stage, and (2) long-term therapy outcomes in subjects that have been treated at a young age. Dystrophin-deficient dogs are considered as one of the best animal models for DMD (McGreevy et al., 2015). However, there are no quantitative data on histopathological and physiological changes in affected aged dogs.

Loss of muscle strength is a primary clinical presentation of DMD patients. Numerous protocols are available for studying muscle physiology in mice. Unfortunately, dystrophin-deficient mdx mice cannot fully recapitulate the contractile features of human patients. For example, the absolute twitch and tetanic muscle forces are maintained at the wild-type level in mdx mice (Watchko et al., 2002). Methods that can faithfully evaluate muscle contractility in canines will be highly valuable for studying physiological changes in dystrophic dogs and evaluating force improvements in preclinical intervention studies. Kornegay et al. (1999) developed an assay to quantify the tetanic isometric torque and the response to eccentric contraction-induced injury at the tibiotarsal joint in hindlimbs. The advantage of this protocol is its non-invasive nature, which allows investigators to follow disease progression and therapeutic response in the same animal over time. Although this is a very useful protocol, it cannot completely meet experimental needs. For example, this protocol measures function from a group of muscles rather than a single muscle. This makes it highly challenging, if not impossible, to correlate the physiological findings with molecular, cellular, biochemical and histological changes in a single muscle. Further, this protocol is not ideal for testing early-stage gene and cell therapies. Owing to the high cost and technical difficulties of vector and cell production, purification and scale-up, often only a limited quantity of test materials is available for early proof-of-principle studies. Such a quantity would not be enough for whole-hindlimb perfusion. Owing to the systemic nature of whole-limb perfusion, the contralateral hindlimb will receive the test materials too (Elverman et al., 2017). This excludes the use of the contralateral muscles as controls in the study.

With this backdrop, we developed a manual *in situ* protocol for studying force generation in a single intact dog muscle in 2012 (Yang et al., 2012). Specifically, we characterized the anatomic (muscle weight, length and physiological cross-sectional area), physiological (absolute and specific tetanic force, force-frequency relationship, and eccentric contraction-induced force reduction) properties of the extensor carpi ulnaris (ECU) muscle in 1.6-year-old affected dogs and age-matched control dogs. In contrast to what has been reported in mdx mice, we found that the absolute tetanic force was significantly reduced in affected dogs (Watchko et al., 2002; Yang et al., 2012). Although our results suggest that the manual protocol we developed is a reliable system for studying muscle contractility in DMD, inherent technical limitations have prevented us from performing comprehensive kinetic analysis. To improve the manual protocol and streamline and standardize the assay, we developed a new automated *in situ* force assay platform and a detailed working protocol for the comprehensive evaluation of canine ECU muscle function.

Given the need to establish the baseline for affected animals that are at the late stage of the disease, we evaluated the ECU muscle in a large cohort of terminal age (3-year-old) affected male dogs (*n*=15) and age/sex-matched normal dogs (*n*=15) using classic anatomical and histopathological methods and our newly developed force assay protocol. The ECU muscle of affected dogs showed significant atrophy and dystrophic pathology. In terms of force measurement, absolute and specific twitch and tetanic forces were significantly decreased, and eccentric contraction-induced force drop was significantly worsened in affected dogs. Kinetic analysis revealed that the time-to-peak tension, half-relaxation time, rate of contraction and rate of relaxation were all significantly reduced in affected dogs. Quantification of the myofiber type revealed an unexpected slow-to-fast fiber-type conversion in the affected ECU muscle. Our findings have laid the groundwork for better utilizing dystrophic canines as a preclinical model for studying DMD pathogenesis and therapy.

## RESULTS

### Development of a novel assay system and assay protocol for the comprehensive evaluation of canine ECU muscle function

**Fig. 1.**
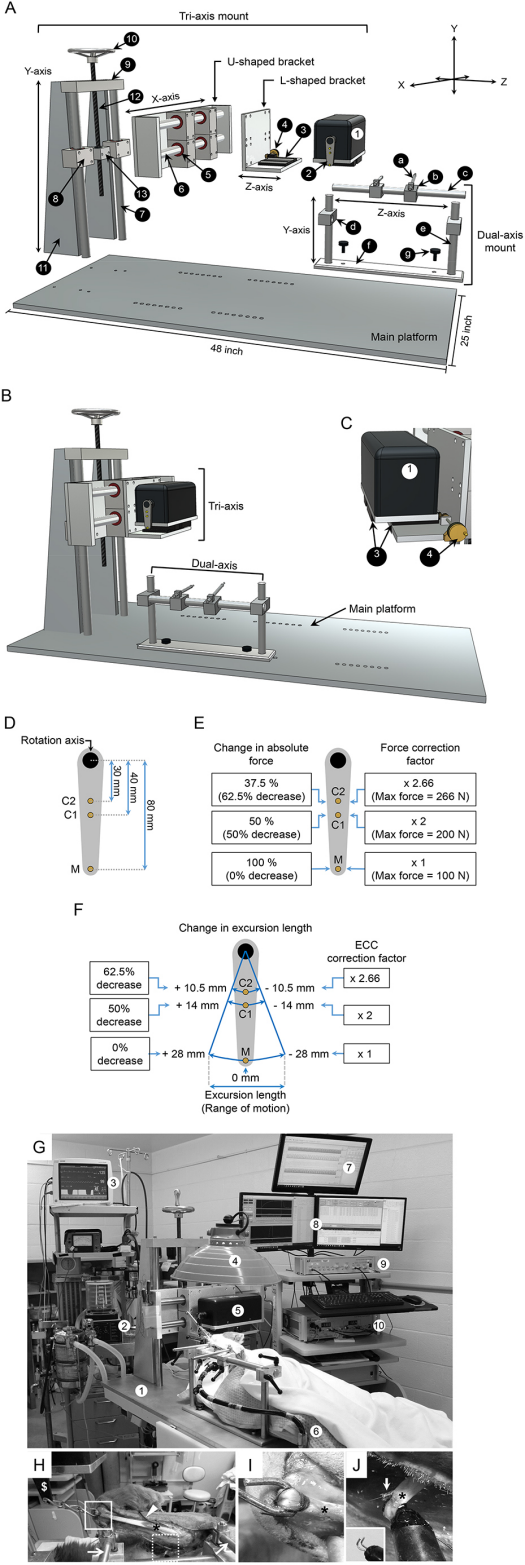
**Muscle force assay system.** (A) A diagrammatic drawing of the disassembled force assay platform. The custom-made muscle force assay platform has three components: a main platform, a tri-axis mount and a dual-axis mount. The numerical number in the tri-axis mount refers to (1) force transducer, (2) lever arm, (3) translational stage, (4) translational knob, (5) linear horizontal bushing, (6) horizontal stainless-steel rod, (7) vertical stainless-steel column, (8) linear vertical bushing, (9) horizontal aluminum plate, (10) adjustment screw with a wheel knob, (11) vertical quadrilateral support plate, (12) threaded steel rod and (13) aluminum mounting adaptor. The small letters in the dual-axis mount refer to (a) bone pin, (b) stainless-steel mount for the bone pin, (c) *x*-axis stainless-steel rod, (d) stainless steel mount module for the *x*-axis stainless-steel rod, (e) vertical stainless-steel rod, (f) bottom plate to attach the dual-axis mount to the main platform and (g) stainless steel screw. (B) A diagrammatic drawing of the assembled force assay platform. (C) A diagrammatic drawing of the L-shaped bracket, force transducer, translational stage and translational knob. The movement of the force transducer on the *x*-axis is regulated by the translation stage and the translation knob. The numbers refer to the same components shown in A. (D) A schematic of the three muscle attachment sites on the lever arm. M refers to the site made by the manufacturer. C1 and C2 refer to the customer engineered sites 1 and 2, respectively. The distance between the center of the rotation axis and the muscle attachment site are marked. (E) The maximum resistant force at each muscle attachment site and the force correction factor. The maximum resistant force at the M, C1 and C2 sites is 100 N, 200 N and 266 N, respectively. The correction factor for the M, C1and C2 sites is 1, 2 and 2.66, respectively. (F) The excursion length at each muscle attachment site and the length correction factor for eccentric contraction (ECC). The excursion length at the M, C1 and C2 sites is ±28 mm, ±14 mm and ±10.5 mm, respectively. (G) A photo of the experiment setting. The numbers refer to (1) the force assay platform, (2) ventilator, (3) vital signs monitor, (4) heat lamp, (5) force transducer, (6) conductive warming blanket, (7) LabChart software for tracing arterial blood pressure and blood flow, (8) Dynamic Muscle Control software, (9) stimulator and (10) force transducer controller module. (H) A photo showing the mounting of the forelimb on the dual-axis mount using the bone pins (arrow). The ECU muscle (asterisk) was surgically exposed and attached at the distal tendon to the force transduce lever arm (dollar sign) through a stainless chain (boxed region). The temperature probe (arrowhead) was placed behind the ECU muscle to monitor the muscle temperature throughout the experiment. The radial nerve was exposed for electric stimulation (dotted boxed region). (I) A closer view of the boxed region in H showing the attachment of the stainless-steel chain to the distal tendon (asterisk) of the ECU muscle. (J) A closer view of the dotted boxed region in H showing the attachment of the radial nerve (asterisk) to the electrode (arrow). The inset shows the electrode without the attached nerve.

The assay system consisted of a modified Aurora Scientific 310C-LR Dual-Mode lever system, a main supporting platform and two custom-made mounting systems (dual-axis mount and tri-axis mount) to allow the seamless integration of muscle dissection, mounting, stimulation, force data acquisition, muscle length control and force analysis (Fig. 1). The dual-axis and tri-axis mounting system provided flexibility to precisely align the force transducer and the ECU muscle irrespective of the dog size and weight (Fig. 1B). The 310C-LR Dual-Mode lever system was designed to measure all dynamic muscle properties during isometric, concentric and eccentric contraction. However, the maximum measurable force can only reach 100 N. To measure a larger force, we engineered C1 and C2, two additional muscle attachment sites, on the lever arm (Fig. 1D-F). This modification extended maximum force measurement to 200 N and 266 N at the C1 and C2 sites, respectively (Fig. 1E).

The assay protocol was developed to accurately quantify the muscle force generated by the ECU muscle from dogs with different body sizes. It included warm up, stimulation condition optimization, tetanic force measurement, twitch force measurement and, finally, ten cycles of eccentric contraction. The warm up was applied to stabilize the muscle for consistent force output during subsequent measurements (Hakim et al., 2011; Sheard et al., 2002). Optimal resting tension, stimulation current, stimulation duration and stimulation frequency were systemically determined for each ECU muscle to achieve the best muscle performance (Table S1).

### Characterization of the experimental subjects and the anatomic properties of the ECU muscle

A total of 15 normal and 15 affected male dogs were included in the study (Table 1). The ECU weight, ECU weight to bodyweight ratio and ECU physiological cross-sectional area (pCSA) were significantly reduced in affected dogs compared to those of normal dogs (Table 1). In both normal and affected dogs, the ECU weight and pCSA correlated significantly with the bodyweight (Fig. S1).

**Table 1.**
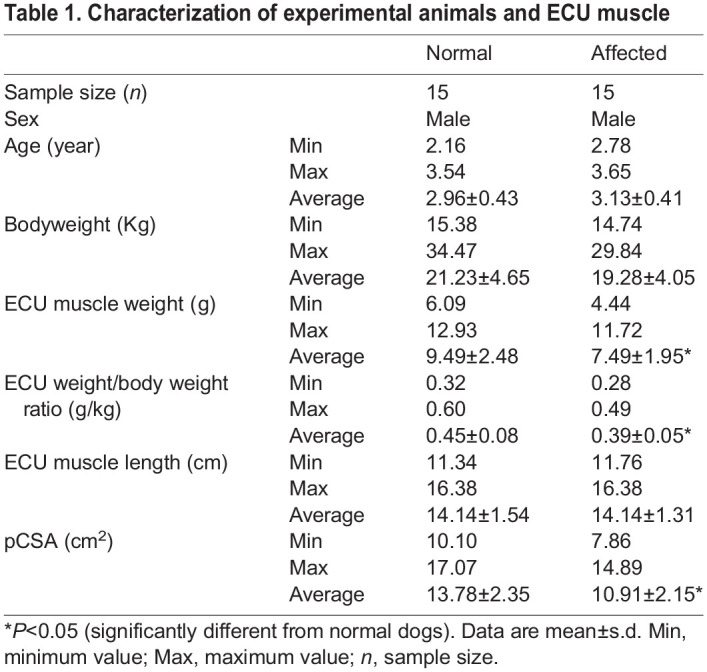
Characterization of experimental animals and ECU muscle

### The ECU muscle of affected dogs showed characteristic dystrophic pathology

**Fig. 2.**
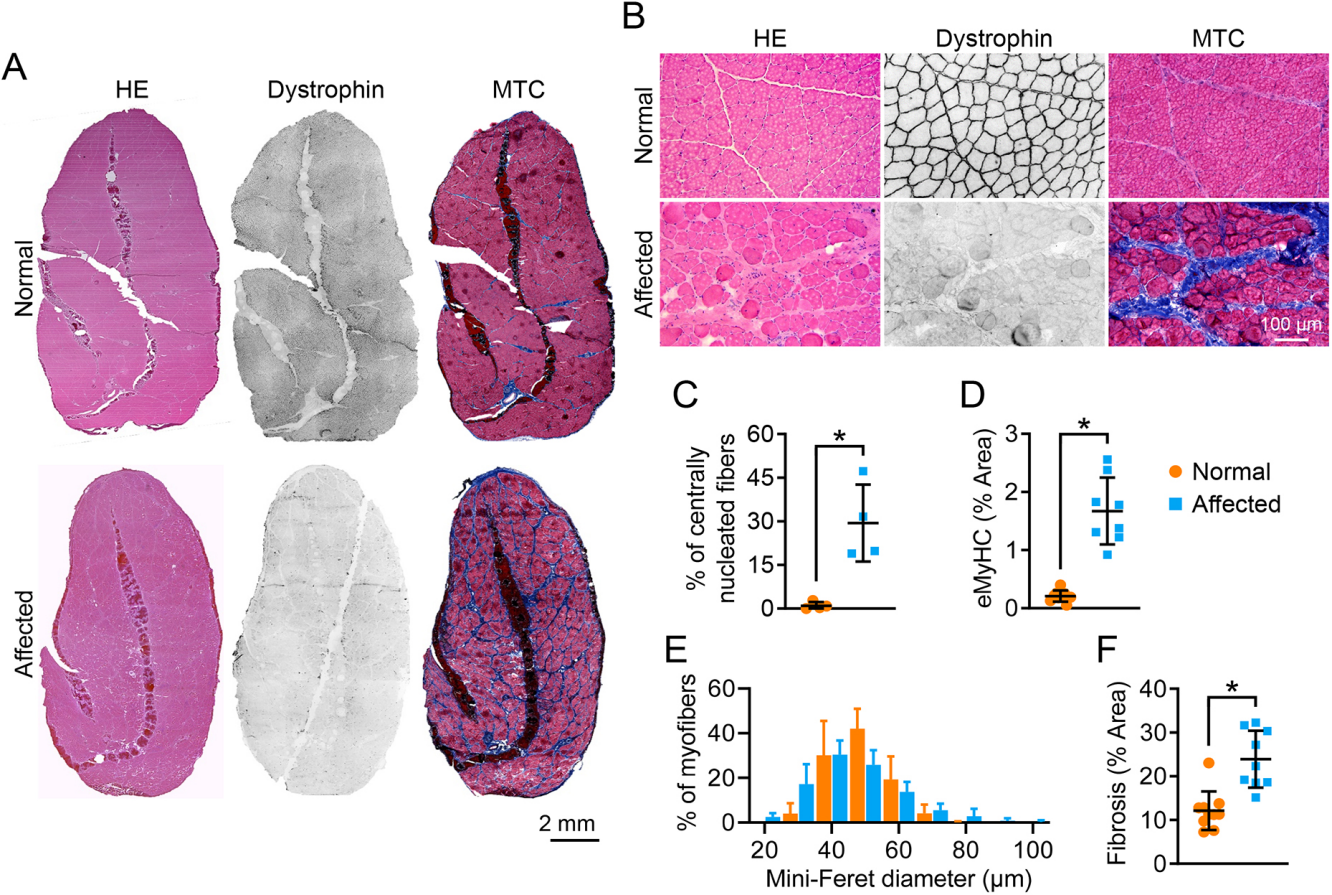
**Dystrophic ECU muscle showed characteristic muscle pathology.** (A) Representative full-view photomicrographs of H&E staining, dystrophin immunostaining and MTC staining from the normal and affected dog ECU muscle. (B) Representative close view photomicrographs of H&E staining, dystrophin immunostaining and MTC staining from normal and affected dog ECU muscle. (C) Quantification of the centrally nucleated myofiber in the normal (*n*=4) and affected dog (*n*=4) ECU muscle. (D) Quantification of the eMyHC-stained myofiber area in the normal (*n*=9) and affected (*n*=8) dog ECU muscle. (E) Morphometric quantification of the myofiber size in the normal (*n*=6) and affected (*n*=7) dog ECU muscle. (F) Percentage of the fibrotic area in the normal (*n*=10) and affected (*n*=9) dog ECU muscle. Data are mean±s.d. *P<0.05 (statistical analysis was performed using unpaired Student’s *t*-test for C, D and F).

As expected, dystrophin expression was detected in the ECU muscle of normal but not affected dogs (Fig. 2). Hematoxylin & Eosin (H&E) staining revealed a uniform fiber size, peripherally located myonuclei and minimal inflammatory cell infiltration in the ECU muscle of normal dogs (Fig. 2A,B). In sharp contrast, the ECU muscle of affected dogs showed abundant centrally localized myonuclei, great myofiber size variation and prominent inflammatory cell infiltration (Fig. 2B). On quantification, ∼30% and <1% of myofibers contained centrally localized nuclei in the affected and normal ECU muscles, respectively (Fig. 2C). Centronucleation marks all regenerated myofibers. To more precisely quantify freshly regenerated myofibers, we performed embryonic myosin heavy chain (eMyHC) staining (Fig. S2). In the normal muscle, eMyHC was barely detected (<0.2%). In the affected muscle, the number of eMyHC^+^ myofibers remained low (∼1.6%) but were significantly higher than that of the normal muscle (Fig. 2D). Myofiber size was quantified using the minimum Feret diameter (Fig. 2E). Extremely large myofibers (mini-Feret diameter ≥80 µm) were only found in the affected ECU muscle (Fig. 2E). Fibrosis is a characteristic feature of dystrophic muscle. Indeed, the fibrotic area in the ECU muscle of affected dogs was significantly larger than that of normal dogs (Fig. 2F).

### Twitch force and twitch contraction kinetics were altered in the affected ECU muscle

**Fig. 3.**
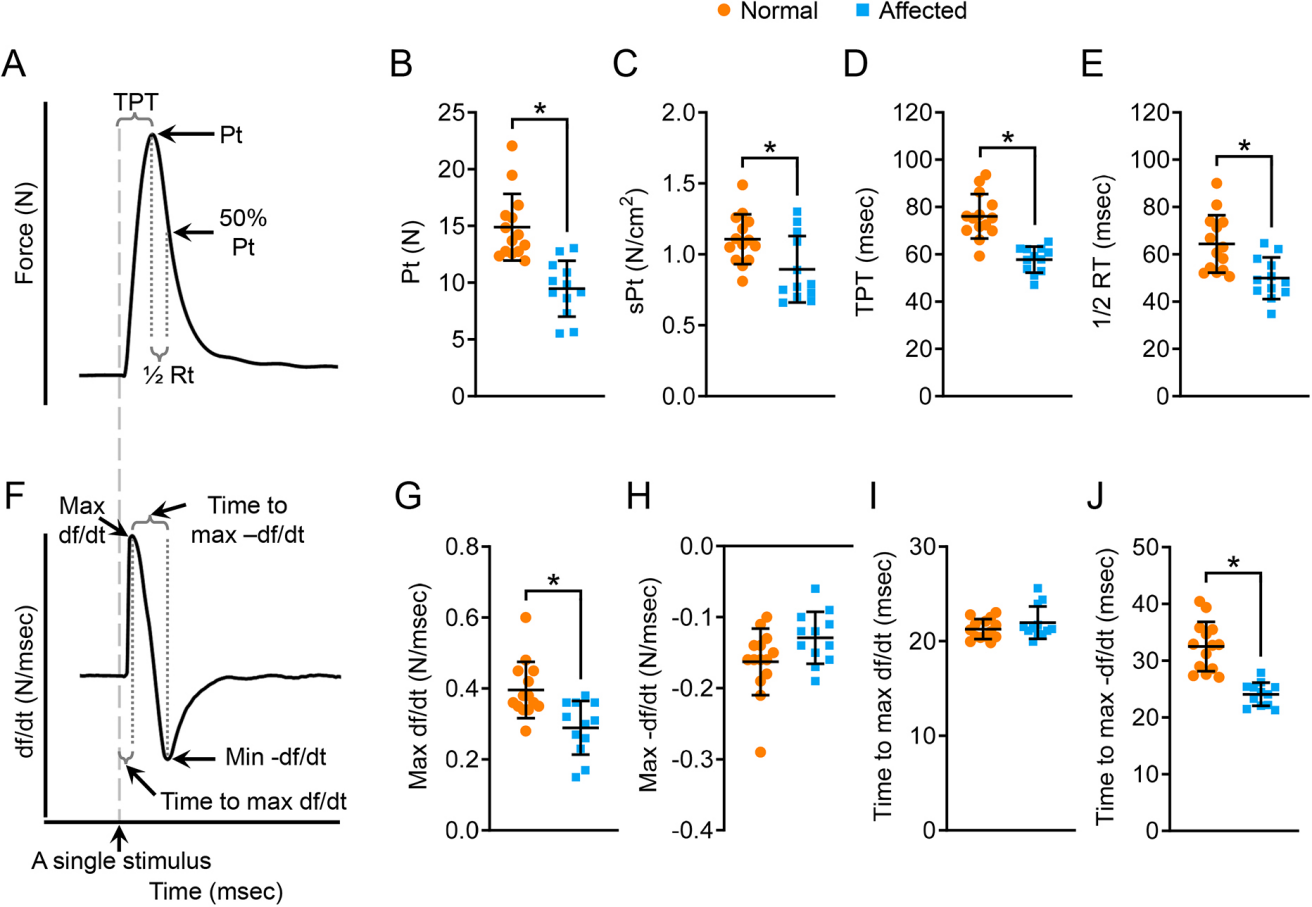
**Characterization of the kinetic properties of twitch contraction.** (A) An illustrative tracing of the force during twitch contraction. Electric stimulation is marked by a gray dashed line. 50% Pt, half absolute twitch force; ½ RT, half-relaxation time (time from Pt to 50% Pt). (B) Quantitative comparison of Pt. (C) Quantitative comparison of sPt. (D) Quantitative comparison of TPT. (E) Quantitative comparison of half-relaxation time. (F) An illustrative tracing of the velocity during twitch contraction. Max df/dt, maximum rate of force development during the contraction phase; max −df/dt, maximum rate of force reduction during the relaxation phase; time-to-max df/dt, time from the start of contraction to mdx df/dt; time-to-max −df/dt, time from Pt to mdx −df/dt. (G) Quantitative comparison of max df/dt. (H) Quantitative comparison of max −df/dt. (I) Quantitative comparison of time-to-max df/dt. (J) Quantitative comparison of time-to-max −df/dt. Sample size: normal (*n*=14) and affected (*n*=12). Data are mean±s.d. *P<0.05 (statistical analysis was performed using the unpaired Student’s *t*-test for B-E and G-J).

We first examined twitch contraction (Fig. 3). Representative force and df/dt tracing of twitch contraction are shown in Fig. 3A and F, respectively. Compared to normal dogs, the absolute twitch force (Pt) and specific twitch force (sPt), time to peak tension (TPT), half-relaxation time, maximum rate of force development (max +df/dt), and time to maximum (time-to-max) −df/dt wer e all significantly reduced in affected dogs (Fig. 3B-E,G,J). The maximum rate of relaxation (max −df/dt) and time-to-max +df/dt showed no difference between normal and affected dogs (Fig. 3H,I).

### Tetanic force and tetanic contraction kinetics were compromised in the affected ECU muscle

**Fig. 4.**
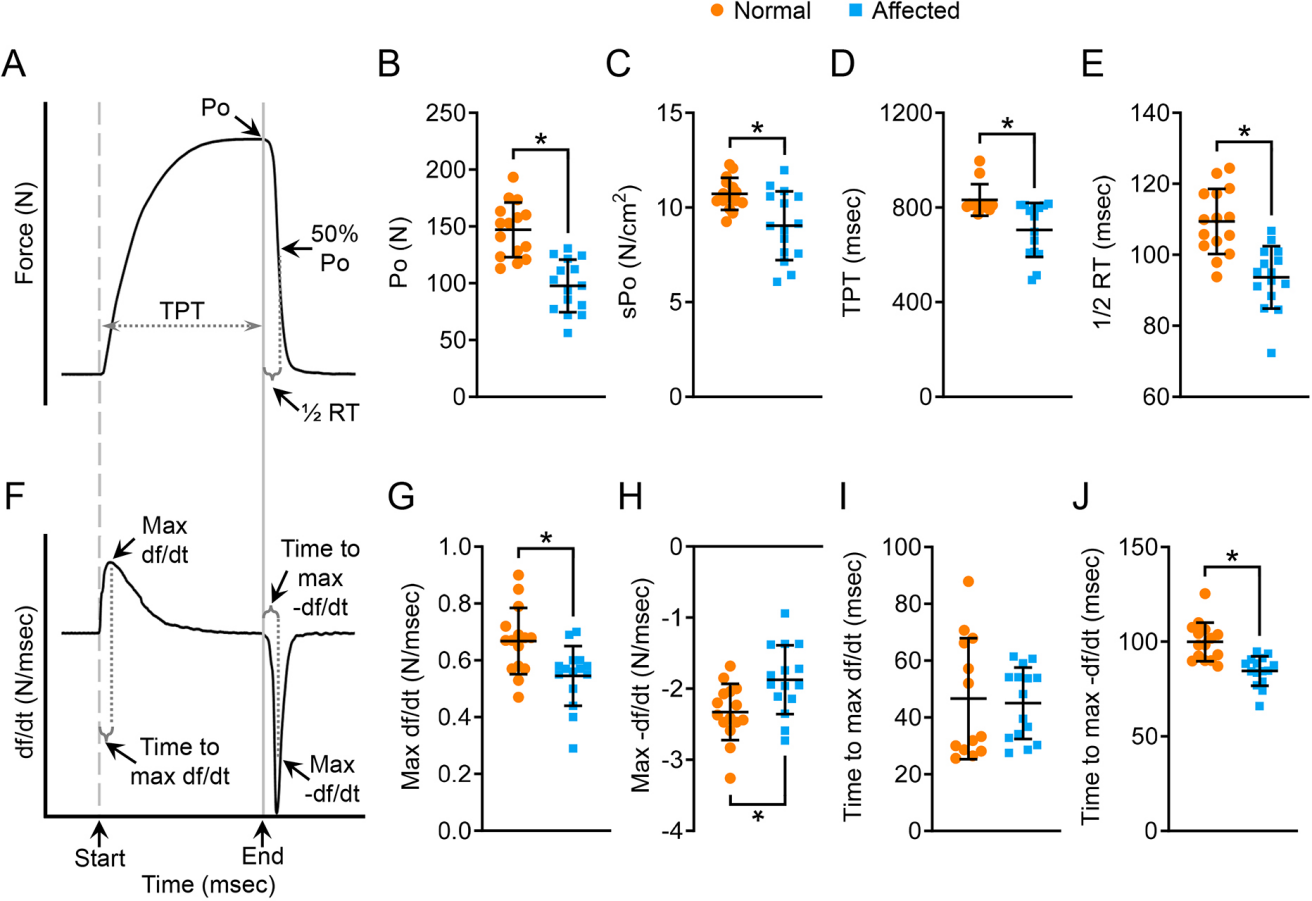
**Characterization of the kinetics properties of tetanic contraction.** (A) An illustrative tracing of the force during isometric tetanic contraction. The start and end of electric stimulation were marked by a gray dashed line and a solid gray line, respectively. 50% Po, half absolute tetanic force. (B) Quantitative comparison of Po. (C) Quantitative comparison of sPo. (D) Quantitative comparison of TPT. (E) Quantitative comparison of half-relaxation time. (F) An illustrative tracing of the velocity during isometric tetanic contraction. Max df/dt, maximum rate of force development during the contraction phase; max −df/dt, maximum rate of force reduction during the relaxation phase; time-to-max df/dt, time from the start of contraction to mdx df/dt; time-to-max −df/dt, time from the end of electric stimulation to mdx −df/dt. (G) Quantitative comparison of max df/dt. (H) Quantitative comparison of max −df/dt. (I) Quantitative comparison of time-to-max df/dt. (J) Quantitative comparison of time-to-max −df/dt. Sample size: normal (*n*=15) and affected (*n*=15). Data are mean±s.d. *P<0.05 (statistical analysis was performed using unpaired Student’s *t*-test for B-E and G-J).

**Fig. 5.**
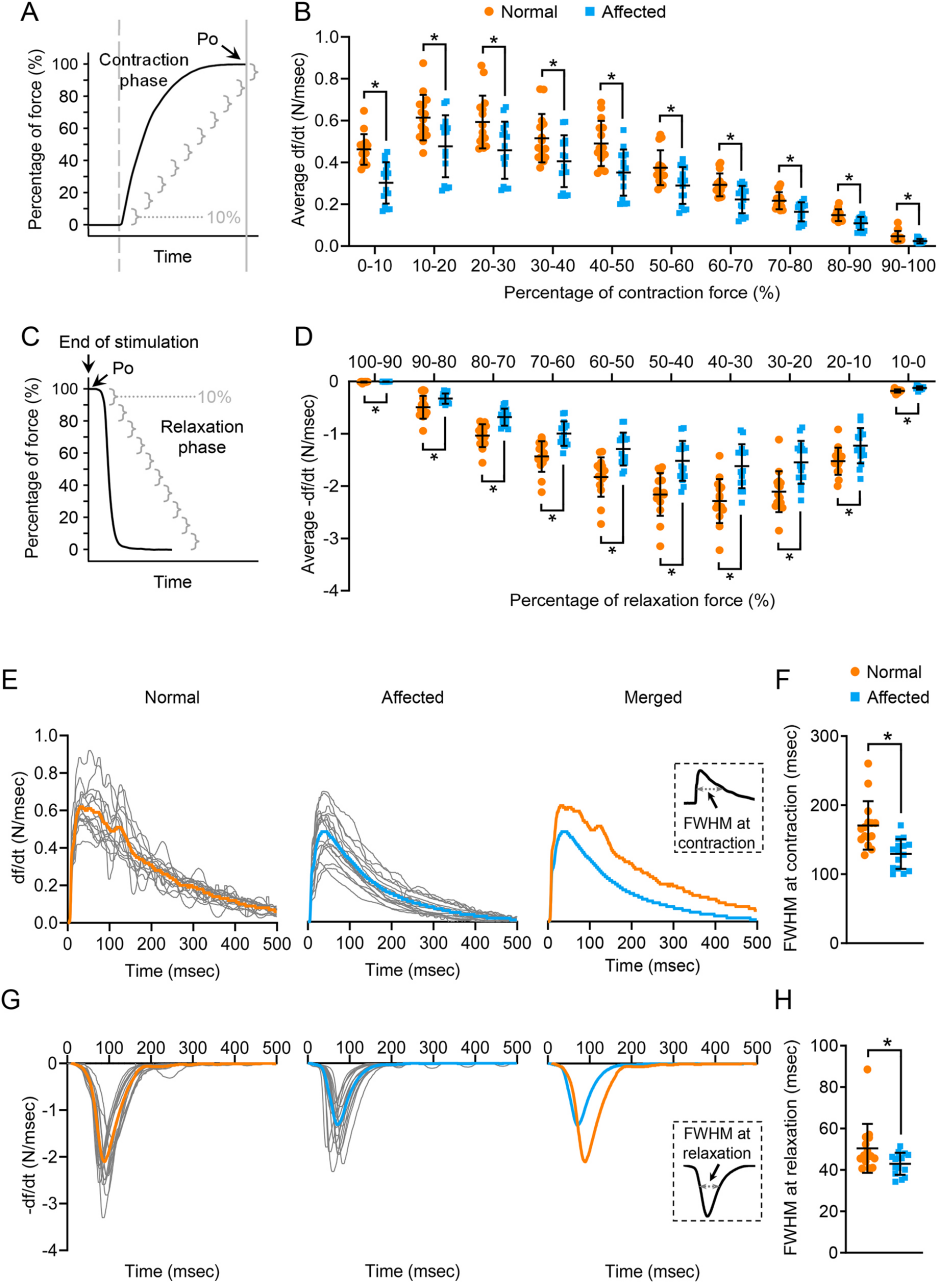
**Characterization of the segmental rate of force development during contraction, segmental rate of force reduction during relaxation, real-time rate of force development during contraction and real-time rate of force reduction during relaxation in isometric tetanic contraction.** (A) An illustrative tracing of the force development during tetanic contraction. The contraction phase refers to the time from the start of electric stimulation (gray dashed line) to the end of electric stimulation (gray solid line). The contraction phase is divided into ten segments (gray brackets). Each segment represents 10% of the force. (B) Average df/dt in every 10% increment of the Po during the contraction phase. (C) An illustrative tracing of the force during the relaxation phase of tetanic contraction. (D) Average −df/dt in every 10% reduction of the Po during relaxation. (E) Real-time tracing of the rate of force development during the contraction phase of tetanic contraction in the normal and dystrophic ECU muscle. gray, tracing from the individual ECU muscle; orange, tracing of the mean real-time rate of force development in the normal ECU muscle; blue, tracing of the mean real-time rate of force development in the dystrophic ECU muscle. The inset shows a diagrammatic illustration of the FWHM at contraction. (F) Quantitative comparison of FWHM at contraction. (G) Real-time tracing of the rate of force reduction during the relaxation phase of tetanic contraction in the normal and dystrophic ECU muscle. Gray, tracing from the individual ECU muscle; orange, tracing of the mean real-time rate of force reduction in the normal ECU muscle; blue, tracing of the mean real-time rate of force reduction in the dystrophic ECU muscle. The inset shows a diagrammatic illustration showing the FWHM at relaxation. (H) Quantitative comparison of FWHM at relaxation. Sample size: normal (*n*=15) and affected (*n*=15). Data are mean±s.d. *P<0.05 (statistical analysis was performed using multiple unpaired *t*-tests for B and D, and unpaired Student's *t*-test for F and H).

Next, we examined tetanic contraction (Figs 4, 5). Representative force and df/dt tracing of tetanic contraction are shown in Fig. 4A and F, respectively. The affected ECU muscle showed a statistically significant reduction of the absolute tetanic force (Po), specific tetanic force (sPo), TPT, half-relaxation time, max +df/dt, max −df/dt and time-to-max −df/dt (Fig. 4B-E,G,H,J). Only the time-to-max +df/dt did not show a significant difference between normal and affected dogs (Fig. 4I).

To study the rate of force development and relaxation more precisely, we quantified the segmental (every 10% of Po) average rate change and real-time rate change (Fig. 5). Segmental df/dt and −df/dt were significantly reduced in the affected ECU muscle during force development and muscle relaxation (Fig. 5A-D). Interestingly, the maximal segmental df/dt difference between normal and affected dogs occurred at the first 10%, and the difference became smaller thereafter (Fig. 5A,B). However, the maximal segmental −df/dt difference between normal and affected dogs occurred when the Po dropped from 50% to 30%. The difference increased gradually when the Po dropped from 100% to 50%, and the difference decreased gradually when the Po dropped from 30% to 0% (Fig. 5C,D). In terms of real-time rate tracing, normal and affected dogs showed similar patterns in df/dt and −df/dt recording (Fig. 5E-H). However, the amplitude of the normal muscle was clearly higher than that of the dystrophic muscle (Fig. 5E,G). To quantitatively compare the real-time df/dt and −df/dt, we calculated the full width at half maximum (FWHM). Compared to normal dogs, the FWHM was significantly reduced during contraction and relaxation in affected dogs (Fig. 5F,H).

### Dystrophin deficiency did not alter the twitch-to-tetanic force ratio

The ratio of the twitch force to the maximum tetanic force is often used to characterize the physiological property of a muscle (Celichowski and Grottel, 1993; Celichowski et al., 2006; Stevens and Faulkner, 2000; Widrick et al., 2016, 2008). In affected dogs, the ratio was 0.11±0.02. In age-matched normal dogs, the ratio was 0.10±0.01. There was no statistically significant difference (Fig. S3).

### Absolute and specific isometric tetanic forces were significantly reduced across a broad range of stimulation frequencies

**Fig. 6.**
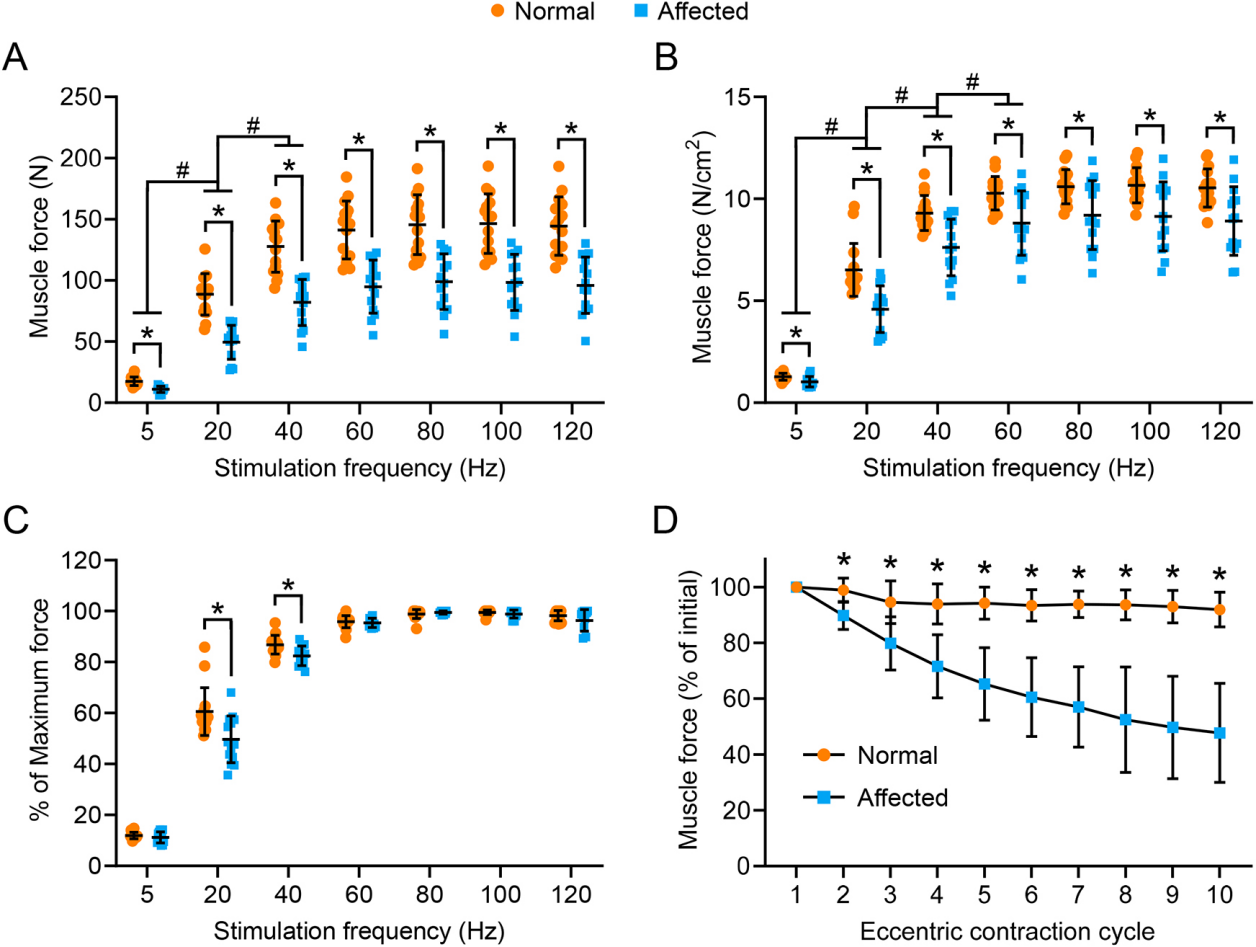
**The force-frequency relationship and eccentric contraction profile in normal and affected ECU muscle.** (A) The absolute isometric force of the ECU muscle at 5, 20, 40, 60, 80, 100 and 120 Hz from normal and affected dogs. (B) The specific isometric force of the ECU muscle at 5, 20, 40, 60, 80, 100 and 120 Hz from normal and affected dogs. (C) The relative muscle force at the indicated stimulation frequency. Sample size for the force-frequency relationship: normal (*n*=15) and affected (*n*=14). (D) Relative changes of the tetanic force during ten cycles of eccentric contraction. The tetanic force at the beginning of the first cycle of eccentric contraction was designated as 100%. Sample size: normal (*n*=15) and affected (*n*=15). Data are mean±s.d. *P<0.05 (the statistical difference between normal and affected dogs at a fixed stimulation frequency in A-C was analyzed by multiple unpaired *t*-tests; the statistical difference of the same group of dogs (normal dogs as a group, affected dogs as another group) at different stimulation frequencies in A and B was analyzed by one-way ANOVA with Tukey’s post hoc test; the statistical difference between normal and affected dogs at each eccentric contraction cycle was analyzed by multiple unpaired *t*-tests). #, significantly different from each other between two indicated frequencies in dogs of the same category (normal versus normal, affected versus affected).

To comprehensively evaluate force generation, we studied the force-frequency relationship over a broad range of stimulation frequencies (5, 20, 40, 60, 80, 100 and 120 Hz) (Fig. 6). In all the frequencies we tested, the ECU muscle of affected dogs generated significantly lower forces (Fig. 6A). In both normal and affected dogs, the force generated at 5 and 20 Hz was significantly lower than the force generated at 20 and 40 Hz, respectively (Fig. 6A). After normalization with the pCSA, the specific force of the affected dog ECU muscle remained significantly lower than normal ECU muscle at all the frequencies (Fig. 6B). In both normal and affected dogs, the specific force generated at 5, 20 and 40 Hz was significantly lower than the specific force generated at 20, 40, and 60 Hz, respectively (Fig. 6B). We also evaluated the force-frequency relationship using relative force (the percentage of the maximum force) (Fig. 6C). There was no difference between normal and affected ECU muscles at 5, 60, 80, 100 and 120 Hz. However, at 20 and 40 Hz, the percent of maximum force generated by the affected ECU muscle was significantly lower than normal ECU muscle (Fig. 6C).

### The ECU muscle of affected dogs was more sensitive to lengthening contraction-induced injury

To characterize contraction-induced muscle damage, we evaluated the percentage of the force decrease through ten cycles of eccentric contraction (Fig. 6D). The accumulative force decrease from the first cycle to the last cycle was merely ∼8% in the normal ECU muscle (Fig. 6D). In sharp contrast, the dystrophic ECU muscle lost ∼55% of the initial force over ten cycles of eccentric contraction (Fig. 6D). A significant difference between normal and affected dogs was observed after every round of eccentric contraction damage (Fig. 6D).

### Affected ECU muscle was mainly composed of type IIa fiber

**Fig. 7.**
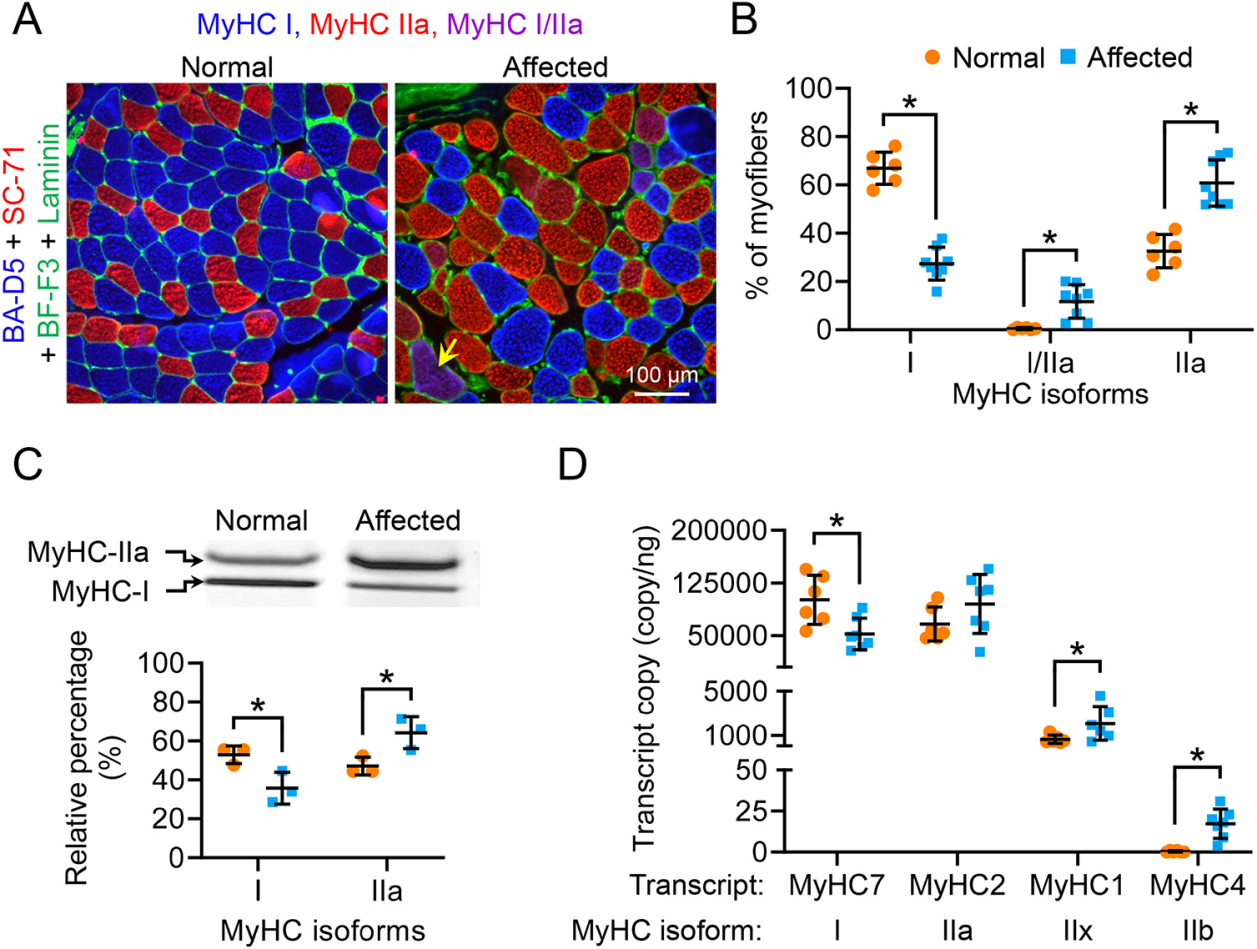
**Affected dog ECU muscle displayed a slow-to-fast myofiber-type switch.** (A) Representative MyHC immunostaining photomicrographs of the ECU muscle from a normal and an affected ECU muscle. Blue, type I myofiber; red, type IIa myofiber; magenta (yellow arrow), type I/IIa hybrid myofiber; green, laminin immunostaining. (B) Quantification of fiber-type composition in normal (*n*=6) and dystrophic (*n*=8) ECU muscle by immunofluorescence staining. (C) Electrophoresis separation and quantification of ECU muscle fiber type. Upper panel, representative electrophoresis silver staining image of the MyHC isoforms in normal and dystrophic ECU muscle. Lower panel, quantification of the relative percentage of type I and type IIa isoforms in normal (*n*=3) and dystrophic (*n*=3) ECU muscle. (D) Quantification of skeletal muscle MyHC isoform transcripts in normal (*n*=6) and dystrophic (*n*=7) ECU muscle. Data are mean±s.d. *P<0.05 (statistical analysis was performed using multiple unpaired *t*-tests in B-D).

Compared to the normal ECU muscle, the affected ECU muscle had shorter TPT and half-relaxation time (Fig. 3D,E, Fig. 5D,E). These are typical kinetic features of the fast muscle (Moran et al., 2005). As contractile kinetics are influenced by the fiber type (Maffiuletti et al., 2016; Schiaffino and Reggiani, 2011), we evaluated fiber-type composition in the ECU muscle of normal and affected dogs by immunofluorescence staining, electrophoresis silver staining and droplet digital PCR (Fig. 7). For immunofluorescence staining, we used a co-staining protocol that can simultaneously detect type I, IIa and IIb myosin using antibodies BA-D5, SC-71 and BF-F3, respectively (Fig. 7A; Fig. S4, Table S2; Acevedo and Rivero, 2006; Schiaffino et al., 1989; Smerdu et al., 2005; Štrbenc et al., 2004). Interestingly, we did not see type IIb myofibers in the ECU muscle. Additional staining of the dog extraocular muscle suggests that the failure to see type IIb in the ECU muscle was not due to a bad antibody or technical error (Fig. S4) (Toniolo et al., 2007). Besides type IIa and IIb, fast myofibers also include type IIx. To detect type IIx by immunofluorescence staining, we tried two different antibodies. Antibody BF-35 has been shown to stain for all the fiber types except for IIx in dog muscle (Acevedo and Rivero, 2006; Smerdu et al., 2005). Type IIx fibers were indeed readily identified in the dog latissimus dorsi muscle but not normal or affected ECU muscle (Fig. S5A,B). Antibody 6H1 has been shown to recognize IIx in mouse, rat and human muscle (Bloemberg and Quadrilatero, 2012; Lucas et al., 2000). However, we failed to detect IIx with 6H1 in dog muscle (Fig. S5B). In summary, the ECU muscle primarily consisted of type I and IIa fibers (Fig. 7A; Figs S4, S5). Quantification revealed that the normal ECU was dominated by type I (∼67%), whereas the affected ECU was dominated by type IIa (∼61%), suggesting a slow-to-fast fiber-type switch in dystrophic ECU muscle (Fig. 7B). The fiber-type switch was confirmed by electrophoresis silver staining (Fig. 7C). We also examined MyHC transcript expression by droplet digital PCR (Table S3; Fig. 7D). As expected, type I and IIa transcripts were the most abundant (several logs higher than type IIx and IIb transcripts). Consistent with immunostaining and silver staining results, the normal ECU had significantly more type I transcripts (Fig. 7D). The affected ECU had more type IIa transcripts but this difference was not statistically significant. Nevertheless, the affected ECU showed significantly more type IIx and IIb transcripts (Fig. 7D).

## DISCUSSION

In this study, we describe a novel *in situ* force assay platform and an optimized force assay protocol for studying the contractility of the canine ECU muscle (Fig. 1; Tables S1, S4). Importantly, we examined histopathology, muscle force and contractile kinetics of the ECU muscle of a large cohort of terminal-age male dystrophic dogs and age/sex-matched normal dogs (Figs 2-7, Table 1; Figs S1-S6, Tables S1, S5). We found characteristic muscle pathologies, such as muscle atrophy, myofiber size variation, degeneration/regeneration, inflammation and fibrosis, in the affected ECU muscle (Fig. 2, Table 1; Fig. S2). A physiology assay showed a significant decrease of the absolute and specific twitch and tetanic forces, a significant shortening of the TPT and half-relaxation time, a significant reduction of the contraction and relaxation rate and a significant aggravation of eccentric contraction-induced muscle damage in the affected ECU muscle (Figs 3-6; Fig. S6). Further analysis revealed an unexpected slow-to-fast myofiber-type switch in the affected ECU muscle (Fig. 7; Figs S4, S5).

Accurate measurement of muscle force depends on a robust assay system. We previously reported a manual protocol for studying the force of the ECU muscle *in situ* in alive dogs (Table S4) (Kodippili et al., 2018a; Shin et al., 2013; Yang et al., 2012). However, this protocol is not suitable for studying the kinetics of muscle contraction. To extend our previous findings and to improve and standardize the assay, we developed a new all-in-one automated assay system (Fig. 1). This novel system has several advantages. First, we have made all the components adjustable to meet the need of studying muscles at different anatomic locations or with different sizes. Second, we have developed a protocol to optimize the stimulation parameters for each muscle (Table S1). This allows the muscle to reach the best performance. For example, in our previous study in 1.6-year-old dogs, the tetanic force of the affected ECU muscle only reached ∼55 N (Table S3) (Yang et al., 2012). In the current study in 3-year-old terminal-age affected dogs, the tetanic force of the ECU muscle reached ∼98 N (Fig. 4B; Table S4). Given aging-associated disease progression in DMD, we expect muscle force to go down at 3 years of age in affected dogs (Lynch et al., 2001). However, our data showed exactly the opposite, suggesting that we had underestimated the muscle force in our previous study. Third, we have improved the accuracy of the eccentric contraction assay. This assay requires forced stretching of a contracting muscle. The lower the variance of the stretch rate, the more accurate the assay. In our previous study, the variance of the stretch rate was 3.99 and 2.98 in normal and affected ECU muscles, respectively (Yang et al., 2012). In the current study, the variance of the stretch rate was reduced to 0.59 and 0.41 in normal and affected ECU muscles, respectively (Table S5).

The average lifespan of dystrophin-deficient dogs is ∼3 years (McGreevy et al., 2015). Quantitative muscle pathology and force data from aged dystrophic dogs would be very useful to guide the design of long-term preclinical intervention studies or studies aimed at testing experimental therapeutics in subjects at the advanced disease stage. To this end, we examined histopathology, anatomy and function of the ECU muscle in 3-year-old male affected dogs (*n*=15) and age/sex-matched normal dogs (*n*=15). The absence of dystrophin expression in the affected ECU muscle was confirmed by immunostaining (Fig. 2A,B). Upon histological examination, we observed typical dystrophic changes, such as inflammation, fibrosis, myofiber size variation and centronucleation in the affected but not normal ECU muscle (Fig. 2). We also observed low-level freshly regenerated myofibers in the affected ECU muscle, suggesting old dystrophic muscle does not have robust acute muscle regeneration (Fig. 2D; Fig. S2).

We previously observed a statistically insignificant reduction of the ECU muscle weight and pCSA in 1.6-year-old affected dogs (Table S4) (Yang et al., 2012). Additionally, the muscle-to-body weight ratio was not altered in those young adult affected dogs (Table S4) (Yang et al., 2012). On the contrary, in terminal-age affected dogs, the muscle weight, pCSA and muscle-to-body weight ratio were all significantly reduced, suggesting muscle atrophy in old dystrophic dogs (Table 1; Table S4). Collectively, the anatomical data confirm the progression of muscular dystrophy in old affected dogs.

Equipped with the new force assay platform and protocol (Fig. 1), we evaluated ECU muscle function (Figs 3-6; Table S3). We first examined muscle force. In twitch contraction, tetanic contraction and force-frequency assays, the absolute force and pCSA-normalized specific force were all significantly reduced in the affected ECU muscle (Figs 3B,C, Fig. 4B,C, Fig. 6A-C; Table S4). These results confirm and expand our previous findings in 1.6-year-old affected dogs (Yang et al., 2012). Together, these studies suggest that force generation capacities are significantly compromised in the ECU muscle of affected dogs.

Next, we evaluated force changes following repeated rounds of eccentric contraction (Fig. 6D). We have previously shown that the ECU muscle in 1.6-year-old affected dogs is highly sensitive to eccentric contraction-induced injury (Kodippili et al., 2018a,b; Shin et al., 2013; Yang et al., 2012). Consistent with these earlier observations, we found a progressive reduction of the force in the ECU muscle of aged affected dogs during repeated cycles of eccentric contraction, and a nominal loss was observed in the ECU muscle of age/sex-matched normal control dogs (Fig. 6D).

Numerous studies have evaluated contractile kinetics in normal and dystrophin-deficient mice (Addinsall et al., 2020; Chan and Head, 2010; Hakim and Duan, 2012, 2013; Hakim et al., 2019; Peczkowski et al., 2020; Rezvani et al., 1995). However, little is known about the kinetics of canine muscle contraction. Using our new system, we examined the ECU muscle contraction kinetics in normal and affected dogs. In both twitch and tetanic contraction, we noticed a significant reduction of the TPT and half-relaxation time (Figs 3, 4). Given that small TPT and half-relaxation time are characteristic features of fast muscle (Moran et al., 2005), we reason that the affected ECU muscle may largely consist of fast myofibers. This rationale has two issues. First, besides a low TPT and half-relaxation time, fast muscle also yields a higher force and has a higher contraction and relaxation rate (Moran et al., 2005). However, this is not the case in the affected ECU muscle. The force, contraction rate and relaxation rate were all significantly reduced in the affected ECU muscle compared to those of the normal ECU muscle (Figs 3–6; Table S3). Second, it is well established that the dystrophic muscle undergoes a fast-to-slow, rather than a slow-to-fast, transition (Fink et al., 1990; Head et al., 1992; Marini et al., 1991; Pedemonte et al., 1999; Webster et al., 1988; Yuasa et al., 2008).

The force reduction is not surprising in a dystrophic muscle because dystrophin-null muscle cells undergo necrosis and are replaced by fatty fibrotic tissue that does not have contractile machinery. The reduction of the contraction (or relaxation) rate, we suspect, may relate to the disproportional change of the force and contraction (or relaxation) time. The rate of contraction (or relaxation) is directly proportional to the force but inversely proportional to the time. For example, during tetanic contraction, the peak force and TPT of normal ECU muscle were ∼147 N and ∼831 ms, respectively (Fig. 4B,E). This yielded an average tetanic contraction rate of ∼0.18 N/ms for normal muscle (Fig. S6B). The peak force and TPT of the affected ECU muscle were ∼100 N and ∼714 ms, respectively (Fig. 4B,E). This yielded an average tetanic contraction rate of ∼0.14 N/ms for dystrophic muscle. This is significantly lower than that of the normal muscle (Fig. S6B). Similarly, the average twitch contraction rate, twitch relaxation rate (based on half-relaxation time) and tetanic relaxation rate (based on half-relaxation time) were all significantly reduced in the affected ECU muscle (Fig. S6).

Based on the literature, we expect a fast-to-slow fiber-type switch in dystrophic muscle. However, based on the TPT and half-relaxation time (Figs 3, 4), we predict a slow-to-fast fiber-type switch in the affected ECU muscle. To resolve the apparent discrepancy, we profiled the myofiber type composition by immunofluorescence staining, electrophoresis and droplet digital PCR (Fig. 7; Figs S4, S5). We found that the dog ECU muscle had no type IIx and IIb fibers (Figs S4, S5). Importantly, we found the affected ECU muscle mainly consisted of the fast type IIa fiber, whereas the normal ECU muscle mainly consisted of the slow type I fiber (Fig. 7). Collectively, we demonstrate for the first time that a dystrophic muscle could undergo a slow-to-fast fiber-type transition. Myofiber type composition is determined by many factors, such as age, nerve activity, exercise, hormones and disease (Schiaffino and Reggiani, 2011). Slow-to-fast fiber-type remodeling has never been observed in dystrophin-deficient muscle. We currently do not have an explanation for the unexpected fiber-type switch in the affected ECU muscle. Future studies are needed to determine whether this is unique to the canine ECU muscle, and more importantly, to understand the molecular mechanism(s) and pathophysiological implications of the slow-to-fast transition observed in our study.

In summary, we have developed a robust assay to comprehensively study the physiology of a single muscle in large mammals. We have also characterized the pathological and contractile changes of the ECU muscle in terminal-age dystrophic dogs. The unexpected discovery of the slow-to-fast myofiber-type transition in the affected ECU muscle highlights the complexity of muscle remodeling in DMD. Our study has paved the way to thoroughly study disease in a single muscle in large animal models.

## MATERIALS AND METHODS

### Experimental dogs

All animal experiments were approved by the Animal Care and Use Committee of the University of Missouri, and were performed in accordance with National Institutes of Health guidelines. All animal experiments were conducted at the University of Missouri. A total of 30 dogs were used in the study, including 15 normal and 15 affected dogs (Table 1). All experimental dogs were male. All experimental dogs were on a mixed genetic background of the golden retriever, Labrador retriever, beagle and Welsh corgi, and were generated in-house by artificial insemination. The genotype of the affected dogs was determined by PCR (Fine et al., 2011; Hakim et al., 2021; Smith et al., 2011). All experimental dogs were housed in an American Association for Accreditation of Laboratory Animal Care-accredited, limited access, conventional animal care facility and kept under a 12-h light/12-h dark cycle. Affected dogs were housed in a raised platform kennel, whereas normal dogs were housed in a regular floor kennel. Depending on the age and size, two or more dogs were housed together to promote socialization. Normal dogs were fed with dry Purina Laboratory Canine Diet 5006 (LabDiet, St Louis, MO, USA, 0001324), whereas affected dogs were fed with wet Purina Proplan Puppy food, as instructed by the veterinarian (Purina Pro Plan Puppy dry and canned food, 38100-02773). Dogs were given *ad libitum* access to clean drinking water. Toys were allowed in the kennel with dogs for activity enrichment. Dogs were monitored daily by the caregivers for overall health condition and activity. A complete physical examination was performed by the veterinarian from the Office of Animal Research at the University of Missouri for any unusual changes in behavior, activity, food and water consumption, and clinical symptoms. The bodyweights of the dogs were measured periodically to monitor growth and body condition. Experimental subjects were euthanized at the end of the study according to the 2013 American Veterinary Medical Association Guidelines for the Euthanasia of Animals.

### Sample size, randomization and blinding

The sample size was not determined by power analysis. Assays were performed on all dogs that were available. No dog was excluded from the analysis. Animals were allocated to the normal and affected groups based on the genotype. No method of randomization was used. All physiological assays were performed without blinding because affected animals were readily identifiable by their dystrophic appearance. For morphometric, biochemical and molecular analyses, each slide (tissue) was assigned a slide (tissue) number. The investigators who performed the assay and quantification were blinded to the animal information.

### Muscle force assay platform

The custom-made muscle force assay platform was designed by C.H.H. to accommodate canines with different body weights and sizes (Fig. 1; Yang et al., 2012). The platform was manufactured using aluminum and stainless steel materials at the University of Missouri Physics Machine Shop (University of Missouri, Columbia, MO, USA). The entire setup had three components: a main platform, a tri-axis mount and a dual-axis mount (Fig. 1). The main platform provided support for the entire system. The tri-axis mount held the force transducer. The dual-axis mount secured the forelimb (Fig. 1). The main platform (25×48 inches) was made of a three-quarter-inch aluminum slab (Fig. 1A).

The tri-axis mount was used to control the movement of the force transducer along the *x*-axis, *y*-axis and *z*-axis (Fig. 1A). It was permanently secured to the right side of the main platform and had three subcomponents. The *x*-axis subcomponent included an L-shaped bracket and a translational stage. The L-shaped bracket was made of two aluminum plates attached perpendicularly to each other. The translational stage and knob [Fig. 1A (3 and 4), Fig. 1C (#3 and 4); Areotech, Pittsburgh, PA, USA, 302SPC] were mounted to the horizontal plate of the L-shaped bracket to regulate movement along the *x*-axis. The force transducer [Fig. 1A (1), Fig. 1C (1), Fig. 1G (5); Aurora Scientific, Aurora, ON, Canada] was secured to the translational stage. The *z*-axis subcomponent included a U-shaped bracket, two horizontal stainless-steel rods [Fig. 1A (6)] and four linear horizontal bushings [Fig. 1A (5)]. The U-shaped bracket was made of three aluminum plates with two lateral plates secured perpendicularly to the third plate (Fig. 1A). Inside the U-shaped bracket, two horizontal stainless-steel rods [Fig. 1A (6)] were secured to the lateral aluminum plates, and two linear horizontal bushings [Fig. 1A (5)] were allowed to slide freely on each rod (Fig. 1A). The attachment of the L-shaped bracket to the linear bushings linked the *x*-axis subcomponent with the *z*-axis subcomponent. The movement of the *x*-axis subcomponent along the *z*-axis was achieved by the sliding of the linear bushings along the horizontal stainless-steel rods (two bushings per rod). The sliding was regulated by the locking mechanism on the linear bushing (Fig. 1A,B). The *y*-axis subcomponent included a wheel knob-controlled adjustment screw [Fig. 1A (10)], a threaded steel rod [Fig. 1A (12)], an aluminum mounting adaptor [Fig. 1A(13)], two vertical bushings [Fig. 1A (8)], a horizontal aluminum plate [Fig. 1A (9)], two vertical stainless-steel columns [Fig. 1A (7)] and two vertical trapezium-shape support plates [Fig. 1A (11)]. The two vertical stainless-steel columns were secured on the top to the horizontal aluminum plate and at the bottom to the main platform (Fig. 1A). Each vertical column contained one vertical bushing, which was allowed to slide freely. A threaded hole was made in the horizontal aluminum plate to accept the threaded steel rod. The wheel knob was attached to the top of the threaded steel rod. The aluminum mounting adaptor was mounted to the bottom of the rod. Two vertical trapezium-shaped support plates were added to support and stabilize the *y*-axis subcomponent. The trapezium-shaped support plate was attached at the top to the horizontal aluminum plate and at the bottom to the main platform (Fig. 1A,B). The attachment of the U-shaped bracket to the vertical bushings and the aluminum mounting adaptor linked the *z*-axis subcomponent with the *y*-axis sub-component. The movement of the *z*-axis subcomponent along the *y*-axis was achieved by the sliding of the vertical bushings along the two vertical stainless-steel columns (one bushing per column). The sliding was regulated by the wheel knob-controlled threaded steel rod (Fig. 1A,B). Together, the linear motion in three different axes allowed the alignment of the force transducer with the forelimb.

The dual-axis mount was used to position the forelimb along the *x*-axis and *y*-axis (Fig. 1A,B). It contained two subcomponents. The *x*-axis subcomponent included a horizontal stainless-steel rod [Fig. 1A (c)], two horizontal stainless-steel bone pin mounts [Fig. 1A (b)] and two custom-made stainless-steel bone pins [Fig. 1A (a)]. The bone pin was threaded at the end to allow secure insertion in the bone (Fig. 1A). Two horizontal stainless-steel mounts slide independently on the stainless-steel rod to allow the accurate position of the bone pins based on the forelimb size (Fig. 1A). The *y*-axis subcomponent included an aluminum plate [Fig. 1A (f)], two vertical stainless-steel columns [Fig. 1A (e)], two vertical attachment modules with a locking mechanism [Fig. 1A (d)] and two stainless-steel screws [Fig. 1A (g)]. Two vertical stainless columns were secured at the bottom to the aluminum plate, which could be secured at different positions on the main platform using stainless screws (Fig. 1A,B). The attachment of the horizontal stainless-steel rod to two vertical attachment modules linked the *z*-axis subcomponent with the *y*-axis subcomponent. The sliding of the two vertical attachment modules along the two vertical stainless-steel columns allowed the *z*-axis subcomponent to move up and down along the *y*-axis (Fig. 1A,B).

### Lever arm modification

The lever arm of the force transducer [Fig. 1A (2), Fig. 1D-F, Aurora Scientific, Aurora, ON, Canada, 310C-LR] had one muscle attachment site. We named this site the M site to indicate it was made by the manufacturer. The M site was positioned 80 mm away from the center of the rotation axis at the bottom end of the lever arm [Fig. 1A (2), Fig. 1D). At the M site, the maximum resistant force was set by the manufacturer at 100 N. In our pilot study, we found that the force of the normal ECU muscle often exceeded 100 N. To overcome this hurdle, C.H.H. and H.T.Y. redesigned the lever arm by introducing two more muscle attachment sites (Fig. 1D). The first site was positioned at half the distance (40 mm) between the M site and the rotation axis of the lever arm (Fig. 1D). We named this site C1 to indicate it was the first site made by the customer. The second site was positioned 30 mm away from the rotation axis center of the lever arm (Fig. 1D). We named this site C2 in the paper to indicate it was the second site made by the customer. These modifications allowed us to accurately measure muscle force up to 200 N at the C1 site and 266 N at the C2 site (Fig. 1E).

The Aurora force transducer was designed to study isometric, concentric and eccentric contraction. The lever arm rotated along its axis during concentric and eccentric contraction. The range of the movement was regulated by the position of muscle attachment (Fig. 1D,F). When the muscle was attached at the M site in a standard 310C-LR Aurora force transducer, the range of the movement (lever arm excursion length) was set at 40 mm (i.e. ±20 mm). To meet the needs of studying eccentric contraction in the canine muscle, the excursion length of the lever arm at the M site was set to 56 mm (i.e. ±28 mm) by the manufacturer in our force transducer (Fig. 1F). When the muscle was attached at the C1 and C2 sites, the range of the movement was ±14 mm (50% reduction) and ±10.5 mm (62.5% reduction), respectively (Fig. 1F). During an eccentric contraction, the contracting muscle was stretched to 105% of the muscle length by force. For the muscle that was attached to the M site, the stretch distance was calculated according to the formula: (muscle length)×5%. For the muscle attached to the C1 site, the stretch distance was calculated according to the formula: (muscle length)×5%×2, where 2 was the correction factor (Fig. 1F). For the muscle attached to the C2 site, the stretch distance was calculated according to the formula: (muscle length)×5%×2.66, where 2.66 was the correction factor (Fig. 1F). For example, if an ECU muscle has a length of 100 mm and is attached to the M site, the stretch length of the muscle will be set at 5 mm (100 mm×5%) in the Dynamic Muscle Control (DMC) software interface (see ‘ECU muscle force measurement system and analysis software’ section). In other words, the total muscle length at the end of the stretch will be 105 mm. If this ECU muscle is attached to the C1 site, the stretch length of the muscle will be set at 10 mm (100 mm×5%×2) in the DMC software interface. If this ECU muscle is attached to the C2 site, the stretch length of the muscle will be set at 13.3 mm (100 mm×5%×2.66) in the DMC software interface.

### Anesthesia and surgical preparation

All procedures were performed by the C.H.H. with the assistance of H.T.Y. and J.T. Body hair in the surgical areas was shaved, and skin was disinfected with 70% ethanol. Anesthesia was first induced by intravenous injection of propofol (6 mg/kg), then the subject was intubated, and anesthesia was maintained with 2-4% isoflurane throughout the experiment. The subject was placed on the main platform and positioned in a dorsal recumbency position using foam wedges (Medline, Northfield, IL, USA). Respiration was maintained using a mechanical ventilator (Ohmeda 7000, Ohmeda, Madison, WI, USA) throughout the experiment. The tidal volume was set at 10 ml/min/kg bodyweight, and the breathing rate was set at 12-15 per min to achieve the partial pressure of CO_2_ between 35 and 42 mm Hg. The body temperature was maintained at 37°C using two conductive blankets (Adroit Medical Systems Inc, Loudon, TN, USA) connected to a heated circulating water bath (Fisher Scientific, Hampton, NH, USA). One was placed underneath the animal, and the other was placed on top of the animal throughout the experiment. Heart rate, electrocardiograph, oxygen saturation (SpO_2_), CO_2_, blood pressure and body temperature were monitored with a veterinary vital sign monitor (DRE Waveline Touch, DRE Veterinary, Louisville, KY, USA) throughout the entire experiment. Vital signs, capillary refill time, mucous membrane color, the palpebral reflex and the pedal reflex were recorded every 15 min.

A catheter (BD Insyte Autoguard Shielded IV, 20 G×1.00″, Becton, Dickinson and Company, Franklin Lakes, NJ, USA) was inserted into the saphenous vein for intravenous saline infusion (Vetivex sodium chloride injection solution 0.9%, Dechra Veterinary Products, Overland Park, KS, USA). The infusion rate was set to 4 ml/kg/h. The skin between the medial and lateral sides of the neck was disinfected with 70% ethanol, and a 4-6-cm segment of the right carotid artery was surgically exposed. The proximal end of the artery was tied with a 2-0 braided silk suture (Surgical Specialties Corporation, Wyomissing, PA, USA) to block the blood flow. A small incision was made in the artery, and a silicone tube (OD, 1.8 mm; ID, 1.0 mm) was inserted and advanced to the thoracic aorta to measure the central blood pressure. The tube was then secured to the carotid artery using a 2-0 braided silk suture and the skin incision site was closed with a 4-0 braided silk suture (Surgical Specialties Corporation).

### Surgical procedure to expose the ECU muscle and radial nerve

All procedures were performed by C.H.H. with the assistance of H.T.Y. and J.T. Below, we describe the surgical procedure for studying the left ECU muscle. The same procedure can also be adapted to study the right ECU muscle. The entire procedure had five steps, including (1) placement of the artery blood flow probe, (2) exposure of the ECU muscle, (3) determination of the ECU muscle length, (4) exposure of the radial nerve, and (5) fixation of the forelimb with bone pins.

To place the blood flow probe, the animal was placed in the left lateral recumbency position. The left forelimb arm was then extended and secured with surgical tape. The medial skin above the elbow was disinfected with 70% ethanol, and the brachial artery was surgically exposed. A 3PS transonic flow probe (Transonic Systems, Ithaca, NY, USA) was placed around the brachial artery to measure blood flow. The space between the artery and the probe was filled with electrode gel (Spectra 360, Parker Laboratories., Fairfield, NJ, USA), and the incision site was closed with a 4-0 braided silk suture (Surgical Specialties Corporation).

To expose the ECU muscle, the animal was carefully repositioned on the right lateral side, and the left forelimb was extended. The skin was disinfected with 70% ethanol, and an incision was made on the lateral side of the left forearm to expose the left ECU muscle. The length of the entire ECU preparation (muscle plus tendon) was measured from the proximal tendon insertion at the medial epicondyle of the humerus to the distal tendon insertion at the carpus. We have previously reported that the tendon length is 16% of the length of the entire ECU preparation (Yang et al., 2012). The experimental ECU muscle length was then calculated by subtracting the tendon length from the length of the entire ECU preparation (Table 1).

The forearm incision was slightly extended proximately to expose the triceps brachii lateral muscle to expose the radial nerve. The radial nerve was then carefully exposed by retracting the triceps brachii lateral muscle and the triceps brachii accessory caudally. The nerve was then carefully dissected between the triceps brachii accessory and the brachialis muscle, and tied with a 1-0 braided silk suture (Surgical Specialties Corporation) at the proximal end and cut close to the collateral radial artery.

To secure the forelimb to the dual-axis mount, a stainless-steel bone pin was screwed to the olecranon, and another bone pin was screwed on the radius bone (Fig. 1H). Finally, the distal ECU tendon was cut at the carpus bone insertion and attached to a stainless-steel chain using a size 0 Vicryl suture (Ethicon, Somerville, NJ, USA; Fig. 1I).

### ECU muscle mounting

To mount the ECU muscle to the force transducer, the animal was placed in the dorsal recumbency position and supported with foam wedges on the muscle force assay platform (Fig. 1G). The forelimb and force transducer were then aligned using the dual-axis mount and tri-axis mount, respectively (Fig. 1G,H). To align the forelimb, we first secured bone pins in the corresponding horizontal stainless-steel bone pin mounts. Next, we adjusted the vertical position of the forelimb to match the height of the animal (Fig. 1G).

To align the force transducer, we first secured the stainless-steel chain to one of the muscle attachment sites on the lever arm (note, the other end of the chain was already attached to the distal end of the ECU tendon). For an animal that was less than 14 kg, the chain was usually attached at the M site. For an animal that was more than 18 kg, the chain was usually attached to the C1 or C2 position on the lever arm (Fig. 1D-F). In the context of this study, the chain was usually attached to the C1 site for normal dogs (≥85% cases). The chain was attached to the C2 site in the remaining normal dogs. For affected dogs, the chain was usually (∼60%) attached to the M site. For the remaining affected dogs, the chain was attached to the C1 site (∼40%). Depending on the force produced by the ECU muscle, the attachment position may be changed during the experiment to avoid exceeding the maximum force limit of the force transducer. After securing the chain, the force transducer was aligned based on the position of the forelimb using the tri-axis mount so that the attachment angle between the lever arm and chain was maintained at 100-105° (Fig. 1H). Following the alignment of the forelimb and force transducer, the resting tension of the ECU muscle was set at 150-200 g using the translational knob of the *x*-axis subcomponent in the tri-axis mount [Fig. 1A (4), Fig. 1C (4)].

### ECU muscle temperature regulation

A temperature probe (YSI 400, Yellow Springs Instrument, Yellow Springs, OH, USA) was placed between the ECU muscle and the radius bone to monitor the muscle temperature throughout the experiment (Fig. 1H). The exposed ECU muscle and tendon were covered with a warm wet saline gauze and then covered with Saranwrap to avoid moisture evaporation (Fig. 1G). A conductive warming blanket was placed on top of the animal to maintain the body temperature. A heat lamp was positioned 30-38 cm above the forelimb to maintain the ECU muscle temperature at 37°C throughout the experiment (Fig. 1G).

### Radial nerve stimulation

The distal end of the radial nerve was secured on a bipolar electrode for electrical stimulation (Fig. 1J). Radial nerve stimulation did not cause forelimb movement because the forelimb was tightly secured on the dual-axis mount. However, radial nerve stimulation resulted in paw extension due to the contraction of the forelimb extensor muscles. To prevent paw extension from moving the chain that links the muscle with the lever arm, the paw was gently secured to the main platform using a nylon rope (Uline, Pleasant Prairie, WI, USA).

### Blood flow and central blood pressure measurement

The 3PS transonic flow probe was connected to a TS420 perivascular flowmeter module (Transonic Systems, Ithaca, NY, USA). One end of the MLT0669 blood pressure transducer (ADInstruments, Colorado Springs, CO, USA) was connected to the silicone tube that was inserted into the carotid artery, and the other end of the blood pressure transducer was connected to the FE221 blood pressure amplifier module (ADInstruments). Both blood flow and blood pressure modules were connected to a PowerLab 4/35 (ADInstruments) interfaced with a PC computer. The blood flow and the arterial blood pressure were monitored throughout the experiment using Powerlab data acquisition software (LabChart, v. 8.1.10, ADInstruments).

### ECU force measurement system and analysis software

Muscle force was measured using the 310C-LR Dual-Mode lever system (Aurora Scientific). The system contains a force transducer (Model # 6400, Cambridge technology Inc., Lexington, MA, USA), a force transducer controller module (Model # 310C-LR, Aurora Scientific) and a stimulator (Model # 701A, Aurora Scientific). The system was connected to a PC computer using an interface (Model # 604A, Aurora Scientific), and it was controlled by DMC software (version 5.420, Aurora Scientific). The controller module was configured by the manufacturer for isometric and eccentric contraction. Muscle force was recorded and analyzed using Dynamic Muscle Analysis software (DMA, version 5.321, Aurora Scientific).

### Determination of the optimal resting tension, optimal stimulation current and optimal stimulation duration

Before each experiment, the force transducer was calibrated using calibration weights (Fisher Scientific). After mounting the ECU muscle to the force transducer, the muscle temperature was monitored and allowed to reach 37°C. The ECU muscle was then stimulated three times at 150 Hz and 10 mA for 200 ms with a 60-s rest between each stimulus to warm up the muscle (Hakim et al., 2011; Sheard et al., 2002).

The starting resting tension was set at 150-200 g. To determine the optimal resting tension, the muscle was stimulated at 60 Hz and 10 mA for 500 ms, and muscle force was recorded. After a 60-s rest, the muscle resting tension was increased by 50 to 100 g. The muscle was then restimulated under that same stimulation condition (60 Hz, 10 mA and 500 ms), and the muscle force data were analyzed. The muscle stimulation was repeated with a gradual increase of the resting tension at each cycle until the muscle force reached the highest value, and an additional increase in the resting tension resulted in a force reduction. The resting tension that yielded the highest isometric force was defined as the optimal resting tension and was used in subsequent experiments. In normal dogs, the optimal resting tension varied between 700 and 2000 g, with the majority (∼80%) between 1000 and 2000 g. In affected dogs, the optimal resting tension varied between 300 and 1600 g, with the majority (∼73%) between 600 and 1600 g (Table S1).

To determine the optimal current, the muscle was set at the optimal resting tension determined from above and stimulated at 60 Hz for 500 ms at 10 mA. The muscle force was recorded. After a 60-s rest, the muscle was restimulated under that same stimulation condition (optimal resting tension, 60 Hz, 500 ms), but at a higher current, and the force was recorded. The muscle stimulation was repeated with a gradual increase of the current at each cycle until the muscle force reached the highest value, and an additional increase in the current resulted in a force reduction. The current that produced the highest muscle force was defined as the optimal current and used in subsequent experiments. In normal dogs, the optimal stimulation current varied between 20 to 600 mA, with the majority (∼73%) between 40 and 80 mA. In affected dogs, the optimal current varied between 20 and 300 mA, with the majority (∼80%) between 20 and 80 mA (Table S1).

To determine the optimal stimulation duration, the muscle was set at the optimal resting tension determined from above and stimulated at 60 Hz at the optimal current determined from above. The muscle force was recorded. After a 60-s rest, the muscle was restimulated under that same stimulation condition (optimal resting tension, 60 Hz, optimal current) using a longer stimulation duration, and the force was recorded. The muscle stimulation was repeated with a gradual increase of the stimulation duration at each cycle until the muscle force reached the maximal stable plateau, and an additional increase in the duration resulted in force reduction near the end of the stimulation. The duration that yielded the maximal stable plateau was defined as the optimal stimulation duration and used in subsequent experiments. In all experimental dogs, the optimal stimulation duration varied between 500 and 800 ms. The optimal stimulation duration in 13% and 87% of normal dogs was 500 ms and 800 ms, respectively. The optimal stimulation duration in 13%, 27% and 60% of affected dogs was 500 ms, 600 ms and 800 ms, respectively (Table S1).

### Force-frequency relationship, absolute tetanic and twitch force, and optimal stimulation frequency

To determine the force-frequency relationship, the muscle was set at the optimal resting tension determined from above and stimulated using the optimal current and optimal duration at different frequencies (5, 20, 40, 60, 80, 100 and 120 Hz). The muscle was rested for 1 min between each stimulation. The highest muscle force obtained from the force-frequency curve was defined as the Po. The frequency that yielded the Po was defined as the optimal stimulation frequency. The optimal stimulation frequency in 20%, 53% and 27% of normal dogs was 80 Hz, 100 Hz and 120 Hz, respectively. The optimal stimulation frequency in 40%, 47% and 13% of affected dogs was 80 Hz, 100 Hz and 120 Hz, respectively (Table S1). After a 2-min rest, the muscle was set at the optimal resting tension and stimulated at 1 Hz using the optimal current. The resulting force was defined as the Pt. The twitch force was not measured in one normal dog and three affected dogs. The force-frequence assay was not conducted in one affected dog.

### Eccentric contraction protocol and muscle weight

After 2 min rest, the ECU muscle was subjected to ten repetitive cycles of eccentric contraction. There were two components in each cycle of eccentric contraction. The first component was a tetanic contraction. The muscle was set at the optimal resting tension determined from above and stimulated at the optimal stimulation current, duration and frequency determined from above to achieve stable tetanic contraction. The second component was forced lengthening. At the end of the tetanic contraction, the muscle was continually stimulated at the optimal stimulation current and frequency for 1 s, and at the same time, the muscle was stretched to 105% of the muscle length at the speed of 5% muscle length per second. The muscle was rested for 1 min between two consecutive eccentric cycles.

At the end of the experiment, the subject was euthanized, and the ECU muscle was carefully dissected. The ECU muscle weight was determined and recorded (Table 1).

### ECU muscle force data analysis

The Pt, Po, TPT, half-relaxation time, segmental rate of muscle contraction (the average contraction rate during a defined range of the percentage of muscle contraction), segmental rate of muscle relaxation (average relaxation rate during a defined range of the percentage of muscle relaxation), maximum rate of muscle contraction (+max df/dt), maximum rate of muscle relaxation (max −df/dt), time-to-max +df/dt, and time-to-max −df/dt were determined using DMA software.

The sPt and sPo were calculated by dividing the Pt and Po with the muscle pCSA, respectively. The pCSA was calculated according to the equation: (muscle weight in gram×cos10.03°)/(1.056 g/cm^3^×Lf in cm) (Yang et al., 2012), where 10.03° is the average pennation angle of the ECU muscle (Yang et al., 2012), 1.056 g/cm^3^ is the muscle density (Mendez and Keys, 1960) and Lf is the optimal fiber length. Lf was determined by multiplying the measured muscle length by the fiber length/muscle length ratio. This ratio is 0.0448 for the ECU muscle (Yang et al., 2012).

To determine the real-time rate of muscle contraction and relaxation, the raw muscle force data were extracted from the DMC software (version 5.420) and analyzed with a MATLAB program developed by the G.Y. The real-time rate of force development during contraction and the real-time rate of force reduction during relaxation were computed using the first-order derivative of the force. The obtained data were smoothed using a fifth order Savitzky–Golay finite impulse response smoothing filter with a frame length of 11 ms. The FWHM during muscle contraction and relaxation was determined from the smoothed curve for each test.

The percentage of force drop during eccentric contraction was calculated according to our published protocols (Hakim et al., 2011, 2013; Yang et al., 2012). Specifically, the tetanic force generated during the first part of the first cycle eccentric contraction was defined as 100%. The tetanic forces obtained in each subsequent cycle were used to calculate force drop induced by eccentric contraction. The percentage of force drop was determined according to the formula: force drop %=100×(T1−Tn)/T1, where T1 stood for the tetanic force obtained during the first cycle, and Tn represented the tetanic force obtained during the nth cycle.

### Histological and immunofluorescence staining

Following euthanization, the ECU muscle was carefully dissected out and snap-frozen in liquid nitrogen-cooled isopentane in optimal cutting temperature (OCT) compound (Sakura Finetek, Torrance, CA, USA) for morphological analysis. Cryosections of 10-μm thickness were used for staining. General muscle histopathology was revealed by H&E staining. Fibrosis was examined by Masson trichrome (MTC) staining. Dystrophin was examined by immunofluorescence staining with a monoclonal antibody against the dystrophin C-terminal domain (Dys-2, (Novocastra, Newcastle, UK, NCL-Dys2, clone: Dy8/6C5, lot 6047395, 1:30 dilution; Kodippili et al., 2014). The eMyHC was detected with antibody F1.652, a mouse moncolonal IgG1 [Developmental Studies Hybridoma Bank (DSHB), University of Iowa, Iowa City, IA, USA, F1.652-s, clone: F1.652, 1:250 dilution]. eMyHC was visualized using Alexa Fluor 594-conjugated mouse anti-goat IgG (H+L) (Thermo Fisher Scientific, Waltham, MA, USA, A11020, 1:100 dilution; Fig. S2, Table S2). To classify the muscle fiber type, we performed immunofluorescence staining using a variety of primary antibodies purchased from the DSHB (Table S2). Specifically, type I fibers were detected with antibody BA-D5, a mouse IgG2b monoclonal antibody [DSHB, BA-D5-s, clone: BA-D5, 1:20 dilution (BA-D5 was deposited to the DSHB by S. Schiaffino, University of Padova, Padova, Italy)] (Acevedo and Rivero, 2006; Smerdu et al., 2005). Type IIa fibers were detected with antibody SC-71, a mouse IgG1 monoclonal antibody [DSHB, SC-71-s, clone: SC-71, 1:100 dilution (SC-71 was deposited to the DSHB by S. Schiaffino); Acevedo and Rivero, 2006; Smerdu et al., 2005; Toniolo et al., 2007]. Type IIb fibers were detected with antibody BF-F3, a mouse IgM monoclonal antibody [DSHB, BF-F3-s, clone: BF-F3, 1:40 dilution (BF-F3 was deposited to the DSHB by Schiaffino, S.)] (Toniolo et al., 2007). The above three antibodies were used together in a triple-staining protocol for the simultaneous detection of type I, IIa and IIb myofibers. Two different methods were used to identify type IIx fibers. In one method, we used antibody BF-35, a mouse IgG1 monoclonal antibody [DSHB, BF-35-s, clone: BF-35, 1:50 dilution (BF-35 was deposited to the DSHB by S. Schiaffino)] (Acevedo and Rivero, 2006; Smerdu et al., 2005; Toniolo et al., 2007) (Fig. S5). BF-35 has been shown to stain all MyHC isoforms except type IIx in dog muscle (Acevedo and Rivero, 2006; Smerdu et al., 2005). Indeed, the unstained type IIx fibers were readily identified using the BF-35 antibody (Fig. S5). In another method, we tested antibody 6H1, a mouse IgM monoclonal antibody [DSHB, 6H1-s, clone: 6H1, 1:50 dilution (6H1 was deposited to the DSHB by C. Lucas)]. Antibody 6H1 has been shown to specifically stain type IIx fibers in mouse, rat and human muscles (Bloemberg and Quadrilatero, 2012; Lucas et al., 2000). However, we failed to detect canine type IIx fibers with antibody 6H1 (Fig. S5). The following secondary antibodies were used in immunostaining: Alexa Fluor 350-conjugated goat anti-mouse IgG2b (Thermo Fisher Scientific, A21140, lot #1827991, 1:50 dilution) for the BA-D5 antibody; Alexa Fluor 594-conjugated goat anti-mouse IgG1 (Catalog # A21125, lot #1819892, 1:100 dilution, Thermo Fisher Scientific) for the SC-71 antibody, FITC-conjugated goat anti-mouse IgM (Catalog # 115-095-075, lot #109485, 1:100 dilution, Jackson ImmunoResearch Labs, West Grove, PA, USA) for the BF-F3 antibody; Alexa Fluor 594-conjugated goat anti-mouse IgG1 (Thermo Fisher Scientific, A21125, lot 1819892, 1:100 dilution) for the BF-35 antibody; and Alexa Fluor 488-conjugated goat anti-mouse IgM (Thermo Fisher Scientific, A121042, lot 1010088, 1:100 dilution) for the 6H1 antibody.

To outline individual myofibers, we used an affinity-purified rabbit anti-laminin polyclonal antibody (MilliporeSigma, Burlington, MA, USA, L9393, lot 055M4815V, 1:200 dilution). Laminin was visualized using Alexa Fluor 488-conjugated rabbit anti-goat IgG (H+L) (Thermo Fisher Scientific, A11070, 1:200 dilution).

All antibodies have been validated by the manufacturers. Additional validations were performed in-house in the presence and/or absence of primary and/or secondary antibodies.

### Morphometric quantification

The percentage of centrally nucleated myofibers was determined from five random microscopic fields (200× magnification) of H&E-stained muscle section using Fiji imaging software (https://fiji.sc, National Institutes of Health, Bethesda, MD, USA). The minimum Feret diameters of the myofibers were quantified from five random microscopic fields (200× magnification) of a digitalized laminin immunostaining image using MyoVision automated image analysis software (https://www.uky.edu/chs/center-for-muscle-biology/myovision#Download; Wen et al., 2018). The percentage of the fibrotic area was determined from a full-view photomicrograph of MTC staining using a Fiji imaging software macro (Kennedy et al., 2006).

The percentage of fiber-type isoforms was determined from five random microscopic fields (200× magnification) of the MyHC triple immunostaining/laminin co-stained muscle section using Fiji imaging software. Specifically, a photomicrograph from each muscle section was taken with the UV-2A (blue), TRITC (red) and FITC (green) fluorescence filters. The three pictures were uploaded to Fiji imaging software and stacked to an RGB composite image. Different fiber types were distinguished based on intracellular color. Specifically, blue for type I fiber, red for type IIa fiber, magenta for type I/IIa hybrid fiber and green for type IIb fiber. Each type was manually counted using the cell counter plug-in module in the software. The total number of fibers was calculated from the sum of each individual fiber-type count. The percentage of each fiber type was determined by dividing the individual fiber type count by the total number of fibers. All photomicrographs were taken with a Lecia DFC700 color camera using a Nikon E800 microscope.

### Electrophoresis evaluation of myofiber type

Myosin-enriched muscle lysate was extracted from liquid nitrogen snap-frozen muscle tissue by homogenization in a buffer containing 10% SDS, 1% 0.5 M EDTA and 12.5% 0.5 M Tris-HCl (pH 6.8); supplemented with Halt protease and phosphatase inhibitor cocktail (Thermo Fisher Scientific, 78440). Next, 1-mm-thick 4% stacking/6% separating SDS polyacrylamide mini gels were prepared according to a modified protocol described previously (Talmadge and Roy, 1993). Specifically, 20 ml of 4% stacking gel solution was prepared using 6 ml of 100% glycerol, 2.66 ml of 30% acrylamide:bisacrylamide (50:1), 2.8 ml of 0.5 M Tris (pH 6.7), 0.8 ml of 100 mM EDTA (pH 7.0), 0.8 ml of 10% SDS, 6.72 ml of deionized water, 200 μl of ammonium persulfate and 10 μl of N,N,N,N-tetramethylethylenediamine. Then, 20 ml of 6% separating gel solution was prepared using 6 ml of 100% glycerol, 4 ml of 30% acrylamide:bisacrylamide (50:1), 2.66 ml of 1.5 M Tris (pH 8.8), 2.0 ml of 1 M glycine, 0.8 ml of 10% SDS, 4.33 ml of deionized water, 200 μl of ammonium persulfate and 10 μl N,N,N,N-tetramethylethylenediamine. 300 ng muscle lysate diluted in 2× Laemmli sample buffer (Bio-Rad, Hercules, CA, USA, 1610737EDU), and was electrophoresed at a constant voltage of 70 V for 30 h. The electrophoresed gel was fixed in a solution containing 30% ethanol and 10% acetic acid for 30 min, followed by overnight fixation in 10% glutaraldehyde. The gel was rinsed with a constant flow of deionized water for at least 4 h and then stained with the Pierce Silver Stain Kit (Thermo Fisher Scientific, 24612) according to the manufacturer's instructions. Densitometry quantification was performed using the Fiji imaging software to determine the intensity of each myosin isoform band. The relative percentage of each isoform was calculated using the equation: (band intensity of the specific myosin isoform)/(band intensity of all myosin isoforms)×100%.

### Myosin heavy chain transcript quantification

RNA was extracted from OCT-embedded tissues using an RNeasy Fibrous Tissue kit (Qiagen). The cDNA was generated using a SuperScript IV Kit (Thermo Fisher Scientific) and quantified using a Qubit ssDNA Assay Kit (Thermo Fisher Scientific). Myosin heavy chain transcripts were quantified by digital droplet PCR in a QX200 ddPCR system (Bio-Rad) using ddPCR Supermix for Probes (no dUTP) (Bio-Rad) and custom-designed primers and probes (Table S3). The data were reported as the transcript copy number per ng of cDNA used in the reaction.

### Statistical analysis

All data are biological replicates. Data are mean±s.d.. Data were checked with the Shapiro–Wilk test to confirm normality. To compare the statistical significance between normal and affected dogs (two-group comparison), an unpaired Student's *t*-test was used. To compare between normal and affected dogs in a grouped data set, multiple unpaired *t*-tests were used. The force-frequency relationship data were analyzed using two approaches. The statistical difference between normal and affected dogs at a fixed stimulation frequency was analyzed using Student's *t*-test. The statistical difference of the same group of dogs (normal dogs as a group, affected dogs as another group) at different stimulation frequencies was analyzed using one-way ANOVA with Tukey's post-hoc test. All statistical analyses were performed using GraphPad Prism software version 9.1.1 (GraphPad Software, La Jolla, CA, USA). The difference was considered significant when *P*<0.05. The datasets used and/or analyzed during the current study are presented in Dataset 1.

## Supplementary Material

Supplementary information
