# Supplementary material for: Extensor carpi ulnaris muscle shows unexpected slow-to-fast fiber-type switch in Duchenne muscular dystrophy dogs
Source: Dis Model Mech. 2021 Dec 16;14(12):dmm049006. doi: 10.1242/dmm.049006 (PMC8688408; doi:10.1242/dmm.049006)
Supplement: Supplementary information [file dmm-14-049006-s1.pdf]

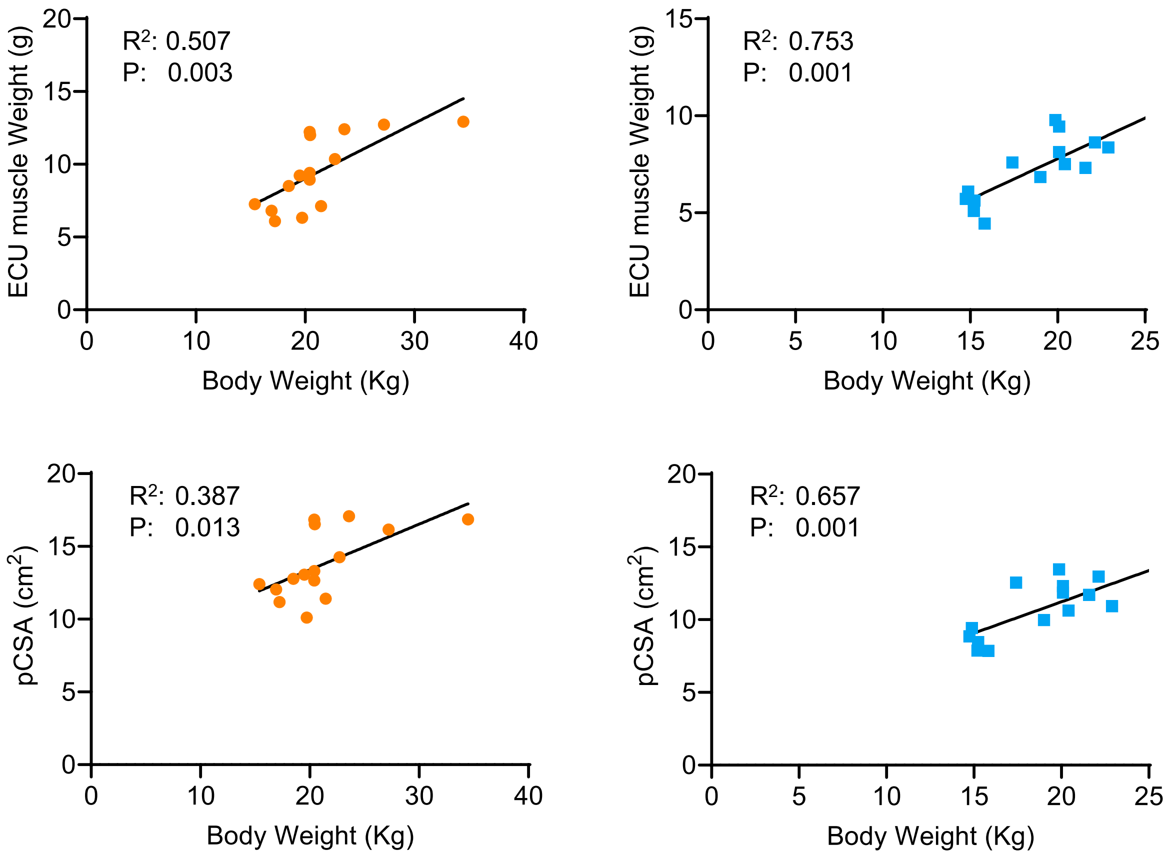

Fig. S1. Correlation between the body weight and the muscle weight, and between the body weight and the physiological cross-sectional area (pCSA) of the extensor carp ulnaris (ECU) muscle in normal (n=15) and affected (n=15) dogs.

Disease Models & Mechanisms • Supplementary information

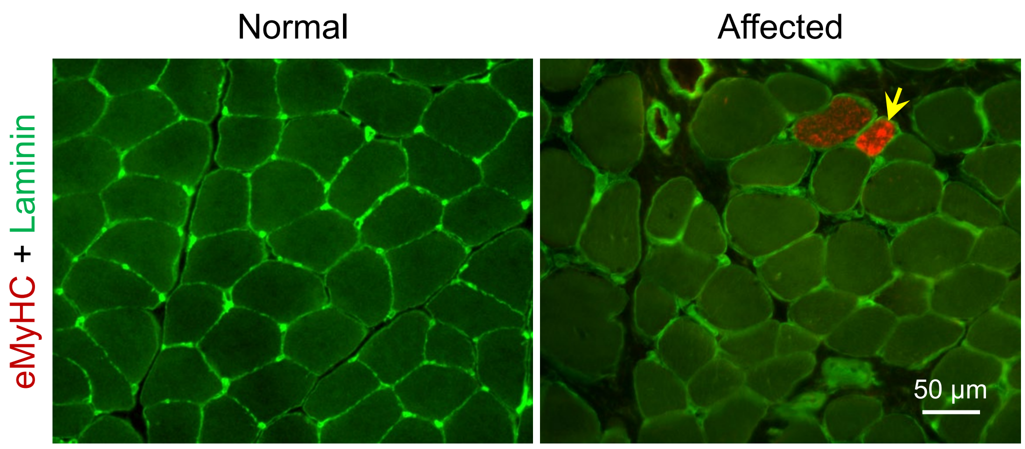

Fig. S2. Affected dog extensor carp ulnaris muscle shows low-level fresh regeneration. Representative embryonic myosin heavy chain (eMyHC) immunofluorescence staining images from normal and affected ECU muscles. Individual myofibers were outlined by laminin immunostaining. Yellow arrow, a myofiber containing eMyHC.

Disease Models & Mechanisms • Supplementary information

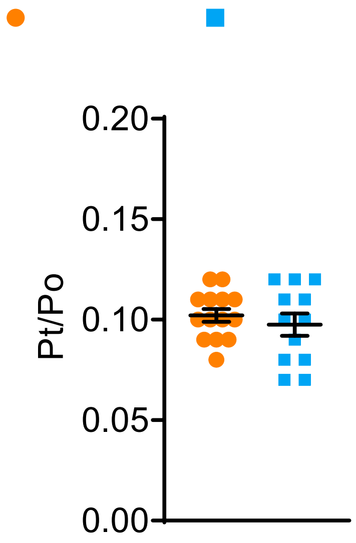

Fig. S3. The twitch (Pt) to tetanic (Po) force ratio in normal (n=14) and affected (n=12) dogs.

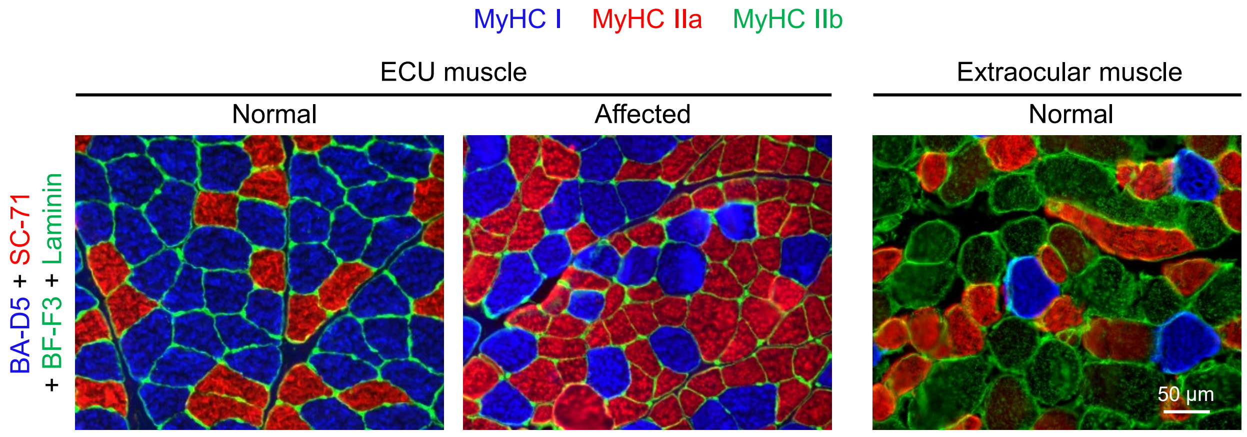

Fig. S4. Type IIb myofiber is absent in the dog ECU muscle. Representative myosin heavy chain isoform (MyHC) immunostaining photomicrographs from normal and affected ECU muscles, and a normal extraocular muscle. Type I myofibers (blue) were stained with antibody BA-D5. Type IIa myofibers (red) were stained with antibody SC-71. Type IIb myofibers (green) were stained with antibody BF-F3. The muscle cell membrane (green) was stained with a polyclonal antibody against laminin.

Disease Models & Mechanisms • Supplementary information

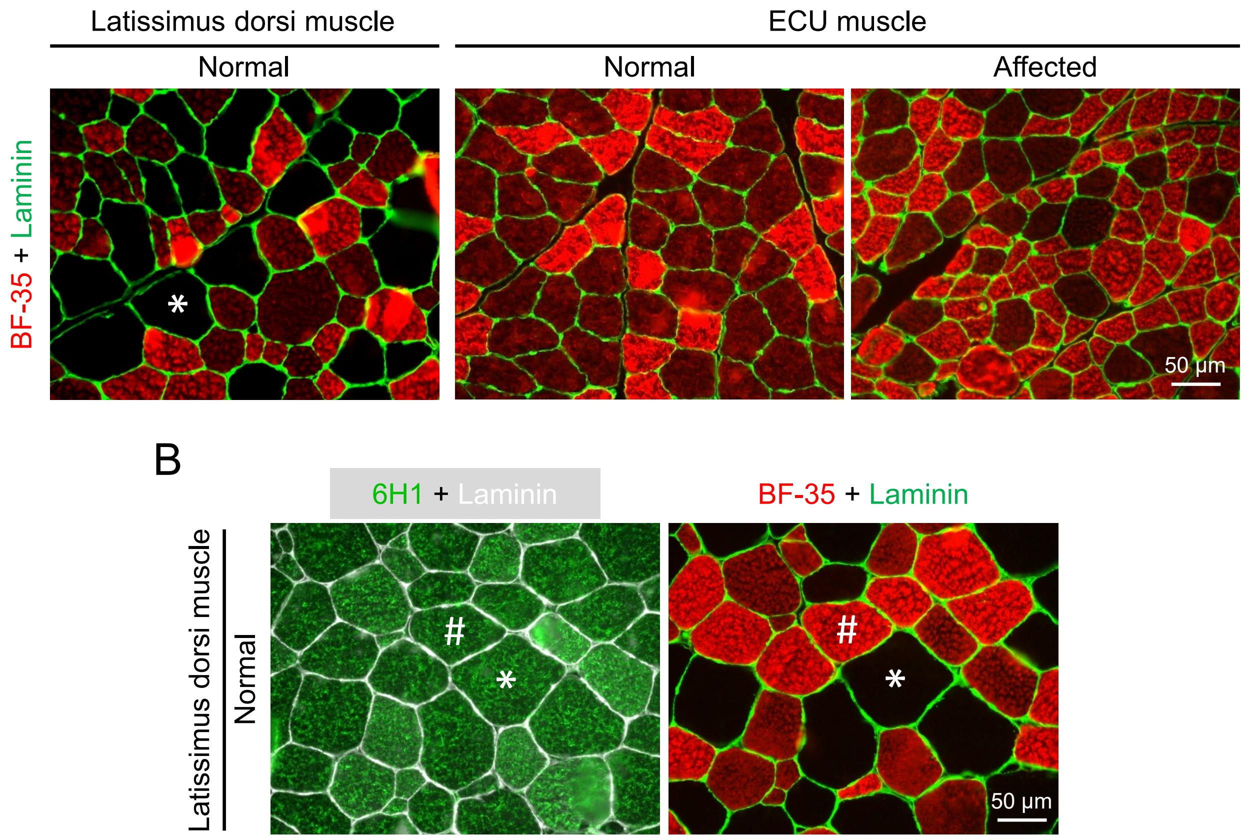

Fig. S5. Evaluation of type IIx fibers in dog muscle. (A) Representative myosin heavy chain isoform immunostaining using antibody BF-35. The BF-35 antibody stains all myosin heavy chain isoforms except type IIx. Type IIx fibers can be readily identified in the latissimus dorsi muscle. No type IIx fiber was detected in the dog ECU muscle. Asterisk (*), a type IIx myofiber. **(B)** Representative myosin heavy chain isoform immunostaining of the latissimus dorsi muscle using antibodies 6H1 and BF-35. The 6H1 antibody cannot identify type IIx myofibers in dog muscle. Asterisk (*), a type IIx myofiber. Pound sign (#), a non-type IIx myofiber.

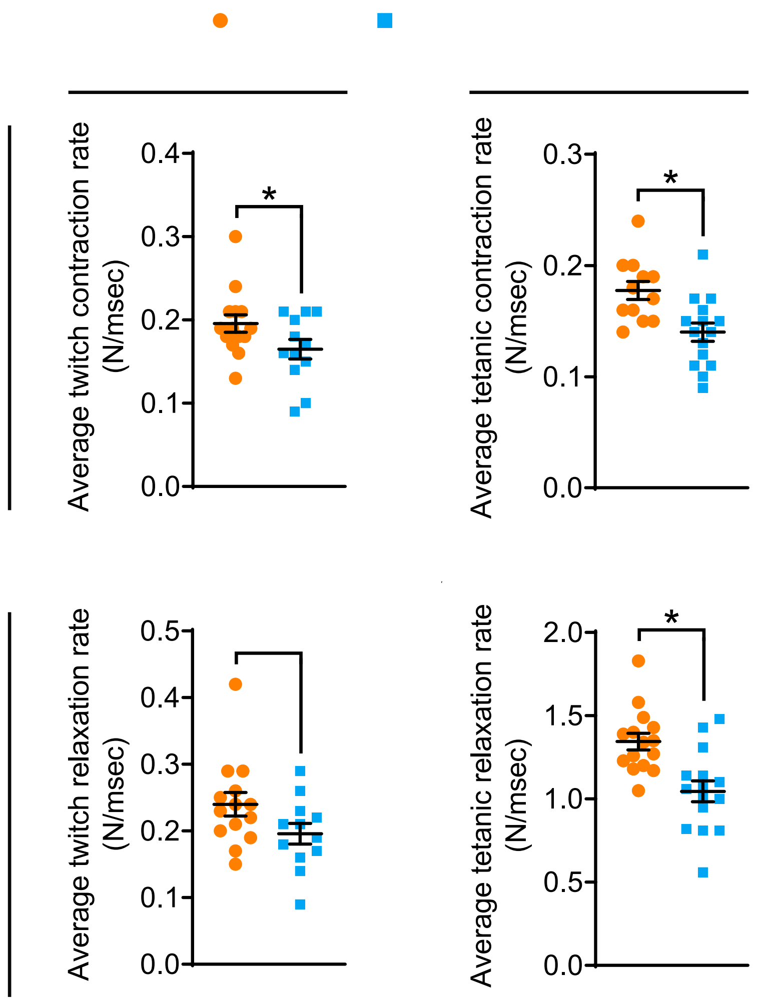

Fig. S6. Quantification of the average contraction and relaxation rate. (A) The average contraction rate of twitch contraction in the normal (n=14) and affected (n=12) ECU muscle. The rate is calculated using the equation Pt/TPT. **(B)** The average contraction rate of tetanic contraction in the normal (n=15) and affected (n=15) ECU muscle. The rate is calculated using the equation Po/TPT. **(C)** The average relaxation rate (based on ½ RT) of twitch contraction in the normal (n=14) and affected (n=12) ECU muscle. The rate is calculated using equation (Pt) / (½ RT). **(D)** The average relaxation rate (based on ½ RT) of tetanic contraction in the normal (n=15) and affected (n=15) ECU muscle. The rate is calculated using equation (Pt) / (½ RT). Asterisk (*), significantly different from each other. $P < 0.05$, unless noted on the graph.

Disease Models & Mechanisms • Supplementary information

Table S1. Optimized stimulation parameters of the ECU muscle

		Normal	Affected
Sample size		15	15
Gender		M	M
Optimal resting tension (g)	Min	700	300
	Max	2000	1600
	Average	1257.53 ± 352.49	728.57 ± 329.17*
	Majority[a]	1000 to 2000 (80%)	600 to 1600 (73%)
Optimal stimulation current (mA)	Min	20	20
	Max	600	300
	Average	100.67 ± 144.54	77.33 ± 77.04
	Majority[a]	40 to 80 (73%)	20 to 80 (80%)
Optimal stimulation duration (msec)	Min	500	500
	Max	800	800
	Average	760.00 ± 105.56	706.67 ± 122.28
	Majority[a]	800 (87%)	800 (60%), 600 (27%)
Optimal stimulation frequency (Hz)	Min	80	80
	Max	120	120
	Average	101.33 ± 14.07	94.67 ± 14.07
	Majority[a]	100 (53%)	100 (47%), 80 (40%)

ECU, extensor carpi ulnaris muscle; Min, minimum value; Max, maximum value.
*, Significantly different from normal dogs.
a, Optimal condition(s) in most of the dogs in the same column.
Data are presented as mean ± SD

Disease Models & Mechanisms • Supplementary information

Table S2. Antibodies used in myofiber type immunofluorescence staining

Antibody name	Isotype	MyHC isoform specificity	Dilution	Figure
BA-D5	IgG2b	MyHC I	1:20	Figs 7, S4
SC-71	IgG1	MyHC IIa	1:100	Figs 7, S4
BF-F3	IgM	MyHC IIb	1:40	Figs 7, S4
6H1	IgM	MyHC IIx	1:50	Fig. S5B
BF-35	IgG1	All isoforms except IIx	1:50	Fig. S5
F1.652	IgG1	eMyHC	1:250	Figs 3D, S2

Table S3. Primers and probes used in droplet digital PCR

Gene	NCBI reference sequence #	MyHC isoform	Forward primer (5' - 3')	Reverse primer (5' - 3')	Probe (5' - 3')
MYH7	NM_001113711.1	I	AGAATGACCTCCAGCTTCAAGTG	AGCGCTCCTCTGCATCAG	AAGCGGAACAAGACAAC
MYH2	NM_001076795.1	IIa	TCAGTGTGGCTAGCATTGATGATC	GTGAGCTTGTAGATGGAGACTTTCT	ACAGATAGTGCTATTGATATTT
MYH1	NM_001113717.1	IIx	ACATTGCCGGCTTTGAGATCTT	GTTTCTCATTGGTGAAGTTGATGCA	CAGGCTGTTAAAATCA
MYH4	NM_001076794.1	IIb	CTGATGAGGGTGGAATTCAAGAAGA	CGGACATTGTACTGGATGCAGAAAA	ATGGAGAGGAGAGAGTCC

Disease Models & Mechanisms • Supplementary information

Table S4. Comparison of the current study and our previous study

		Current study (Hakim *et al.*)		Our previous study (Yang *et al.*)	
		Normal	Affected	Normal	Affected
Sample size		15	15	7	7
Sex		M	M	M	M & F
Age (year)	Min	2.16	2.78	0.74	0.84
	Max	3.54	3.65	2.55	2.52
	Average	2.96 ± 0.43	3.13 ± 0.41	1.64 ± 0.79	1.61 ± 0.78
Body Weight (Kg)	Min	15.38	14.74	13.00	10.90
	Max	34.47	29.84	32.20	22.30
	Average	21.23 ± 4.65	19.28 ± 4.05	20.34 ± 7.29	16.57 ± 4.10
ECU muscle weight (g)	Min	6.09	4.44	3.60	2.95
	Max	12.93	11.72	15.46	10.51
	Average	9.49 ± 2.48	7.49 ± 1.95*	8.37 ± 4.84	6.08 ± 2.47
ECU weight / Body weight ratio (g/kg)	Min	0.32	0.28	0.25	0.27
	Max	0.60	0.49	0.54	0.51
	Average	0.45 ± 0.08	0.39 ± 0.05*	0.39 ± 0.11	0.36 ± 0.09
ECU muscle length (cm)	Min	11.34	11.76	9.70	14.00
	Max	16.38	16.38	19.50	19.00
	Average	14.14 ± 1.54	14.14 ± 1.31	14.48 ± 3.71	16.04 ± 2.13
pCSA (cm^2)	Min	10.10	7.86	7.19	4.39
	Max	17.07	14.89	19.65	11.51
	Average	13.78 ± 2.35	10.91 ± 2.15*	11.37 ± 4.51	7.68 ± 2.16
Po (N)	Min	113.02	56.23	52.80	24.64
	Max	193.38	130.71	159.70	81.80
	Average	147.05 ± 24.11	97.63 ± 21.13*	97.15 ± 43.38	54.94 ± 18.70*
Po / muscle weight (N/g)	Min	12.75	8.86	10.33	7.79
	Max	19.86	16.82	15.54	12.08
	Average	15.95 ± 2.12	13.30 ± 2.41*	12.45 ± 2.16	9.21 ± 1.42*
Po / pCSA (N/cm^2)	Min	9.26	6.08	6.84	5.62
	Max	12.27	11.97	9.81	8.42
	Average	10.72 ± 0.84	9.04 ± 1.82*	8.38 ± 1.08	7.04 ± 0.96*

ECU, extensor carpi ulnaris muscle; M, Male; F, Female; N; newton; Po, tetanic force; pCSA, physiological cross-sectional area; Min, minimum value; Max, maximum value.
*, Significantly different from normal dogs.
Data are presented as mean ± SD

Table S5. Comparison of the parameters used in eccentric contraction in the current study and our previous study

		Current study (Hakim *et al.*)		Our previous study (Yang *et al.*)	
		Normal	Affected	Normal	Affected
% Length stretched	Min	5.00	5.00	4.99	3.13
	Max	5.00	5.00	5.00	5.06
	Average	5.00 ± 0.00	5.00 ± 0.00	5.00 ± 0.00	4.56 ± 0.65
Stretch rate (mm/s)	Min	5.67	5.88	6.58	6.05
	Max	8.19	8.19	10.19	10.35
	Average	7.07 ± 0.77	7.07 ± 0.65	7.90 ± 2.00	7.14 ± 1.72
	Variance	0.59	0.43	3.99	2.98

Min, minimum value; Max, maximum value.
Data are presented as mean ± SD

Dataset 1. Raw data tables

Fig. 2c

% of Centrally nucleated fibers	
**Normal**	**Affected**
0.32	47.26
0.00	31.63
2.81	19.82
0.86	18.98
**Average:** 1.00	29.42
**SD:** 1.26	13.22
**Sample size (n):** 4	4

Statistics Analysis

Test: unpaired *t*-test

P value: 0.0052

Fig. 2D

eMyHC (% Area)	
**Normal**	**Affected**
0.40	1.78
0.22	1.38
0.18	2.38
0.25	1.22
0.26	0.92
0.13	2.56
0.05	1.31
0.21	1.83
0.19	
**Average:** 0.21	1.67
**SD:** 0.10	0.57
**Sample size (n):** 9	8

Statistics Analysis

Test: unpaired *t*-test

P value: <0.0001

Fig. 2E

Mini-Feret diameter (µm)	% of myofibers												
	**Normal**						**Affected**						
**20**	0	0	0	0	0	0	5	1	2	1	2	5	2
**30**	0	7	1	12	2	2	14	15	16	10	14	37	15
**40**	16	38	13	55	28	31	17	34	28	34	35	33	32
**50**	50	34	41	29	48	50	17	31	31	27	29	16	30
**60**	28	16	33	4	20	15	20	10	15	17	14	6	14
**70**	5	5	11	0	2	2	11	6	5	7	4	2	4
**80**	0	0	1	0	0	0	10	2	2	3	1	0	2
**90**	0	0	0	0	0	0	3	0	0	1	0	1	1
**100**	0	0	0	0	0	0	2	0	0	0	0	0	0

Mini-Feret diameter	Normal		Normal	
	**Average**	**SD**	**Average**	**SD**
**20**	0.00	0.00	2.57	1.59
**30**	4.00	4.20	17.29	8.24
**40**	30.17	14.02	30.43	5.88
**50**	42.00	8.14	25.86	6.06
**60**	19.33	9.37	13.71	4.23
**70**	4.17	3.53	5.57	2.66
**80**	0.17	0.37	2.86	3.04
**90**	0.00	0.00	0.86	0.99
**100**	0.00	0.00	0.29	0.70
**Sample size (n):**	6		7	

Fig. 2D

Fibrosis (% Area)	
**Normal**	**Affected**
7.62	18.65
11.30	15.15
7.25	32.30
12.86	31.66
13.76	19.22
23.07	22.33
11.30	18.54
12.82	27.18
9.78	30.34
11.28	
**Average:** 12.10	23.93
**SD:** 4.41	6.52
**Sample size (n):** 10	9

Statistics Analysis

Test: unpaired *t*-test

P value: 0.0002

Fig. 3B	
Pt (N)	
Normal	Affected
19.47	5.65
12.35	12.77
15.73	10.91
14.24	5.53
13.32	9.18
11.93	10.14
12.76	7.31
16.83	13.04
22.06	8.65
12.57	9.13
13.71	9.86
15.92	11.54
14.85	
12.74	

	Normal	Affected
**Average:**	14.89	9.48
**SD:**	2.94	2.45
**Sample size (n):**	14	12

Statistics Analysis
Test: unpaired *t* -test
P value: <0.0001

Fig. 3C	
sPt (N/cm^2)	
Normal	Affected
1.49	0.67
1.11	1.09
1.23	0.89
1.07	0.70
1.05	1.16
0.96	0.75
1.06	0.77
1.18	1.23
1.29	0.79
1.10	0.73
0.81	0.66
0.96	1.30
0.92	
1.26	

	Normal	Affected
**Average:**	1.11	0.90
**SD:**	0.17	0.23
**Sample size (n):**	14	12

Statistics Analysis
Test: unpaired *t* -test
P value: 0.0146

Fig. 3D	
TPT (msec)	
Normal	Affected
93.70	53.87
76.10	62.27
75.40	52.90
75.27	62.43
76.70	65.40
90.90	62.40
69.93	47.07
70.17	60.77
72.47	59.33
66.17	50.97
71.80	57.63
86.37	57.87
80.87	
59.27	

	Normal	Affected
**Average:**	76.08	57.74
**SD:**	9.39	5.50
**Sample size (n):**	14	12

Statistics Analysis
Test: unpaired *t* -test
P value: <0.0001

Fig. 3E	
1/2RT (msec)	
Normal	Affected
66.80	41.41
63.12	49.22
73.99	48.14
60.59	64.68
71.49	58.16
80.91	55.27
54.38	34.78
58.28	44.61
52.38	50.70
52.06	44.04
53.24	45.66
73.59	62.26
90.00	
50.61	

	Normal	Affected
**Average:**	64.39	49.91
**SD:**	12.17	8.81
**Sample size (n):**	14	12

Statistics Analysis
Test: unpaired *t* -test
P value: 0.0022

Fig. 3G	
Max df/dt (N/msec)	
Normal	Affected
0.48	0.17
0.34	0.36
0.45	0.36
0.38	0.15
0.35	0.23
0.28	0.32
0.34	0.26
0.45	0.38
0.60	0.27
0.36	0.31
0.35	0.30
0.38	0.36
0.36	
0.42	

	Normal	Affected
**Average:**	0.40	0.29
**SD:**	0.08	0.08
**Sample size (n):**	14	12

Statistics Analysis
Test: unpaired *t* -test
P value: 0.0019

Fig. 3H	
Max -df/dt (N/msec)	
Normal	Affected
-0.21	-0.09
-0.14	-0.17
-0.14	-0.16
-0.16	-0.06
-0.13	-0.10
-0.10	-0.12
-0.16	-0.14
-0.19	-0.19
-0.29	-0.12
-0.16	-0.14
-0.18	-0.15
-0.15	-0.11
-0.11	
-0.16	

	Normal	Affected
**Average:**	-0.16	-0.13
**SD:**	0.05	0.04
**Sample size (n):**	14	12

Statistics Analysis
Test: unpaired *t* -test
P value: 0.0548

Fig. 3I	
Time to max df/dt (msec)	
Normal	Affected
22.10	23.97
19.85	21.10
20.50	21.73
20.40	24.40
21.20	25.60
23.03	20.80
21.00	21.10
21.70	21.37
20.47	21.50
20.50	19.97
22.53	21.30
21.83	20.73
22.80	
19.97	

	Normal	Affected
**Average:**	21.28	21.96
**SD:**	1.06	1.72
**Sample size (n):**	14	12

Statistics Analysis
Test: unpaired *t* -test
P value: 0.2249

Fig. 3J	
Time to max -df/dt (msec)	
Normal	Affected
39.40	21.27
35.80	25.40
31.20	22.20
27.37	25.30
27.10	24.47
32.93	27.87
32.70	22.10
28.60	24.07
34.70	23.30
32.83	25.33
27.60	21.47
40.47	26.20
35.50	
28.97	

	Normal	Affected
**Average:**	32.51	24.08
**SD:**	4.34	2.06
**Sample size (n):**	14	12

Statistics Analysis
Test: unpaired *t* -test
P value: <0.0001

Fig. 4B	
Po (N)	
Normal	Affected
175.11	77.37
160.13	103.00
120.93	72.02
132.15	125.77
141.04	56.23
152.91	85.63
128.56	121.56
123.38	71.92
157.80	112.45
193.38	130.71
113.02	97.99
163.49	80.62
152.97	124.30
173.49	93.76
117.35	111.13
**Average:** 147.00	97.63
**SD:** 24.12	23.13
**Sample size (n):** 15	15
**Statistics Analysis**	
**Test:** unpaired *t* -test	
**P value:** <0.0001	

Fig. 4C	
sPo (N/cm^2)	
Normal	Affected
10.38	9.15
12.27	8.80
10.82	6.08
10.35	10.22
10.60	7.15
12.08	10.86
10.36	9.03
10.25	7.63
11.06	10.59
11.33	11.97
9.92	7.56
9.71	6.43
9.26	8.35
10.73	10.58
11.62	11.15
**Average:** 10.72	9.04
**SD:** 0.84	1.82
**Sample size (n):** 15	15
**Statistics Analysis**	
**Test:** unpaired *t* -test	
**P value:** 0.0031	

Fig. 4D	
TPT (msec)	
Normal	Affected
814.20	809.00
811.30	608.90
812.80	493.60
809.60	603.80
790.90	612.40
996.80	513.50
801.60	796.20
772.10	660.00
808.90	786.60
804.00	810.50
944.80	815.50
815.10	762.10
	810.60
	698.00
	798.20
**Average:** 831.80	705.30
**SD:** 66.95	114.60
**Sample size (n):** 12	15
**Statistics Analysis**	
**Test:** unpaired *t* -test	
**P value:** 0.0024	

Fig. 4E	
1/2RT (msec)	
Normal	Affected
117.24	95.09
118.68	97.50
102.57	72.35
103.84	84.95
105.49	100.83
110.36	104.17
122.96	106.79
97.89	88.44
110.65	98.35
105.77	91.14
93.84	91.85
117.15	84.52
124.42	95.20
109.91	92.75
100.22	100.89
**Average:** 109.40	93.65
**SD:** 9.20	8.78
**Sample size (n):** 15	15
**Statistics Analysis**	
**Test:** unpaired *t* -test	
**P value:** <0.0001	

Fig. 4G	
Max df/dt (N/msec)	
Normal	Affected
0.72	0.40
0.79	0.57
0.67	0.58
0.65	0.70
0.47	0.29
0.58	0.44
0.57	0.60
0.53	0.55
0.69	0.60
0.90	0.58
0.57	0.50
0.68	0.56
0.67	0.55
0.85	0.69
0.68	0.57
**Average:** 0.67	0.55
**SD:** 0.12	0.10
**Sample size (n):** 15	15
**Statistics Analysis**	
**Test:** unpaired *t* -test	
**P value:** 0.0053	

Fig. 4H	
Max -df/dt (N/msec)	
Normal	Affected
-2.47	-1.37
-2.37	-1.95
-2.00	-1.74
-2.07	-2.35
-2.47	-0.94
-2.44	-1.37
-1.85	-2.14
-2.23	-1.38
-2.59	-1.94
-3.26	-2.73
-2.20	-2.11
-2.41	-1.72
-1.68	-2.59
-2.83	-1.81
-2.04	-1.96
**Average:** -2.33	-1.87
**SD:** 0.39	0.48
**Sample size (n):** 15	15
**Statistics Analysis**	
**Test:** unpaired *t* -test	
**P value:** 0.0089	

Fig. 4I	
Time to max df/dt (msec)	
Normal	Affected
28.60	54.20
26.50	39.60
87.90	28.60
28.10	36.70
30.00	54.20
52.00	53.90
33.30	27.50
57.20	32.80
31.90	48.20
67.90	59.10
66.20	61.50
70.70	30.30
25.60	60.70
	34.20
	53.80
**Average:** 46.61	45.02
**SD:** 21.30	12.56
**Sample size (n):** 13	15
**Statistics Analysis**	
**Test:** unpaired *t* -test	
**P value:** 0.809	

Fig. 4J	
Time to max -df/dt (msec)	
Normal	Affected
104.30	84.40
106.50	88.50
89.80	66.00
93.10	74.20
92.40	91.40
98.30	93.80
110.00	94.80
90.10	78.90
101.90	88.40
98.40	83.60
87.10	84.70
107.50	77.00
125.40	87.50
103.70	84.50
90.00	90.60
**Average:** 99.90	84.55
**SD:** 10.21	7.81
**Sample size (n):** 15	15
**Statistics Analysis**	
**Test:** unpaired *t* -test	
**P value:** <0.0001	

Fig. 5B

Average df/dt (N/msec)

% of contraction force	Normal															Affected														
	Dog # 1	Dog # 2	Dog # 3	Dog # 4	Dog # 5	Dog # 6	Dog # 7	Dog # 8	Dog # 9	Dog # 10	Dog # 11	Dog # 12	Dog # 13	Dog # 14	Dog # 15	Dog # 1	Dog # 2	Dog # 3	Dog # 4	Dog # 5	Dog # 6	Dog # 7	Dog # 8	Dog # 9	Dog # 10	Dog # 11	Dog # 12	Dog # 13	Dog # 14	Dog # 15
0-10	0.50	0.56	0.40	0.48	0.37	0.44	0.41	0.39	0.48	0.65	0.39	0.46	0.44	0.49	0.48	0.24	0.36	0.35	0.45	0.17	0.25	0.43	0.33	0.39	0.19	0.27	0.35	0.18	0.41	0.18
10-20	0.70	0.67	0.52	0.57	0.45	0.55	0.50	0.53	0.68	0.87	0.56	0.64	0.63	0.74	0.60	0.40	0.56	0.57	0.69	0.28	0.43	0.57	0.55	0.60	0.29	0.45	0.54	0.27	0.68	0.28
20-30	0.68	0.58	0.47	0.54	0.47	0.52	0.48	0.50	0.63	0.86	0.52	0.65	0.66	0.83	0.50	0.39	0.52	0.54	0.65	0.28	0.42	0.56	0.53	0.58	0.28	0.48	0.43	0.27	0.66	0.27
30-40	0.57	0.50	0.42	0.46	0.44	0.42	0.37	0.47	0.52	0.75	0.41	0.58	0.60	0.75	0.47	0.35	0.43	0.50	0.59	0.24	0.38	0.49	0.49	0.50	0.25	0.43	0.35	0.24	0.59	0.24
40-50	0.69	0.42	0.57	0.56	0.35	0.42	0.45	0.36	0.47	0.66	0.37	0.47	0.53	0.61	0.44	0.28	0.37	0.44	0.55	0.20	0.35	0.43	0.40	0.43	0.22	0.38	0.33	0.20	0.48	0.20
50-60	0.53	0.34	0.29	0.34	0.30	0.34	0.31	0.27	0.38	0.52	0.34	0.39	0.38	0.51	0.37	0.24	0.31	0.38	0.42	0.16	0.29	0.39	0.31	0.34	0.19	0.34	0.24	0.18	0.39	0.17
60-70	0.39	0.27	0.23	0.31	0.25	0.29	0.28	0.23	0.29	0.37	0.27	0.30	0.28	0.40	0.23	0.19	0.24	0.28	0.31	0.12	0.22	0.30	0.26	0.25	0.15	0.26	0.19	0.14	0.29	0.13
70-80	0.30	0.19	0.17	0.23	0.19	0.22	0.21	0.18	0.23	0.28	0.17	0.23	0.20	0.28	0.19	0.14	0.19	0.21	0.20	0.09	0.16	0.23	0.19	0.19	0.11	0.20	0.15	0.11	0.19	0.10
80-90	0.21	0.13	0.11	0.17	0.13	0.15	0.16	0.12	0.15	0.18	0.12	0.15	0.13	0.19	0.13	0.09	0.13	0.14	0.15	0.06	0.11	0.14	0.12	0.13	0.07	0.13	0.10	0.07	0.13	0.06
90-100	0.11	0.05	0.03	0.08	0.04	0.04	0.08	0.03	0.03	0.05	0.03	0.04	0.04	0.03	0.03	0.02	0.04	0.03	0.05	0.02	0.04	0.03	0.02	0.03	0.01	0.02	0.02	0.01	0.02	0.01
**Sample size (n):**								15															15							

Statistics Analysis

Test: unpaired *t*-test

% of contraction force	Normal		Affected	
	Average	SD	Average	SD
0-10	0.46	0.07	0.30	0.10
10-20	0.61	0.11	0.48	0.15
20-30	0.59	0.13	0.46	0.14
30-40	0.52	0.12	0.41	0.12
40-50	0.49	0.11	0.35	0.11
50-60	0.37	0.08	0.29	0.09
60-70	0.29	0.05	0.22	0.07
70-80	0.22	0.04	0.16	0.05
80-90	0.15	0.03	0.11	0.03
90-100	0.05	0.02	0.02	0.01

% of contraction force	P Value
0-10	0.0003
10-20	0.0288
20-30	0.0288
30-40	0.0288
40-50	0.0128
50-60	0.0288
60-70	0.0182
70-80	0.0155
80-90	0.0093
90-100	0.0155

Fig. 5D

Average -df/dt (N/msec)

% of relaxation force	Normal															Affected														
	Dog # 1	Dog # 2	Dog # 3	Dog # 4	Dog # 5	Dog # 6	Dog # 7	Dog # 8	Dog # 9	Dog # 10	Dog # 11	Dog # 12	Dog # 13	Dog # 14	Dog # 15	Dog # 1	Dog # 2	Dog # 3	Dog # 4	Dog # 5	Dog # 6	Dog # 7	Dog # 8	Dog # 9	Dog # 10	Dog # 11	Dog # 12	Dog # 13	Dog # 14	Dog # 15
0-10	0.00	-0.02	-0.01	-0.01	-0.01	-0.01	-0.04	0.00	-0.01	-0.01	0.00	-0.01	0.00	-0.01	-0.01	0.00	0.00	-0.01	0.00	0.00	0.00	-0.01	0.00	0.00	0.00	-0.01	-0.01	0.00	0.00	0.00
10-20	-0.59	-0.64	-0.45	-0.18	-0.16	-0.18	-0.41	-0.44	-0.67	-0.94	-0.64	-0.32	-0.65	-0.57	-0.54	-0.28	-0.42	-0.45	-0.36	-0.18	-0.25	-0.42	-0.40	-0.40	-0.17	-0.41	-0.31	-0.24	-0.41	-0.20
20-30	-1.11	-1.19	-0.87	-0.79	-0.76	-0.77	-0.85	-0.94	-1.18	-1.55	-1.05	-1.06	-1.14	-1.29	-0.96	-0.64	-0.79	-0.82	-0.75	-0.41	-0.61	-0.90	-0.68	-0.86	-0.53	-0.92	-0.63	-0.51	-0.74	-0.43
30-40	-1.45	-1.50	-1.06	-1.17	-1.19	-1.24	-1.10	-1.37	-1.60	-2.12	-1.47	-1.55	-1.49	-1.92	-1.29	-0.89	-1.13	-1.16	-1.23	-0.60	-0.90	-1.23	-0.88	-1.21	-0.84	-1.37	-1.02	-0.78	-1.09	-0.61
40-50	-1.84	-1.81	-1.34	-1.55	-1.62	-1.70	-1.35	-1.81	-2.07	-2.72	-1.86	-1.98	-1.66	-2.46	-1.62	-1.10	-1.51	-1.46	-1.72	-0.77	-1.12	-1.55	-1.09	-1.54	-1.11	-1.76	-1.38	-1.04	-1.45	-0.77
50-60	-2.24	-2.16	-1.72	-1.90	-2.09	-2.16	-1.66	-2.12	-2.46	-3.15	-2.13	-2.30	-1.64	-2.78	-1.89	-1.27	-1.81	-1.66	-2.11	-0.89	-1.29	-1.89	-1.27	-1.81	-1.29	-2.02	-1.62	-1.22	-1.71	-0.91
60-70	-2.45	-2.36	-1.96	-2.06	-2.40	-2.41	-1.83	-2.21	-2.58	-3.23	-2.18	-2.40	-1.42	-2.77	-2.02	-1.36	-1.94	-1.73	-2.32	-0.94	-1.36	-2.10	-1.37	-1.93	-1.36	-2.10	-1.71	-1.29	-1.81	-0.97
70-80	-2.32	-2.22	-1.93	-1.91	-2.42	-2.32	-1.72	-2.01	-2.32	-2.85	-1.99	-2.16	-1.11	-2.38	-1.94	-1.32	-1.84	-1.63	-2.27	-0.88	-1.29	-2.08	-1.32	-1.83	-1.28	-1.98	-1.62	-1.22	-1.70	-0.94
80-90	-1.71	-1.59	-1.51	-1.33	-2.00	-1.72	-1.25	-1.42	-1.54	-1.89	-1.43	-1.46	-0.92	-1.56	-1.53	-1.08	-1.42	-1.31	-1.86	-0.67	-1.02	-1.69	-1.06	-1.41	-1.01	-1.58	-1.29	-0.96	-1.32	-0.74
90-100	-0.19	-0.18	-0.16	-0.16	-0.17	-0.19	-0.12	-0.17	-0.19	-0.25	-0.16	-0.19	-0.20	-0.22	-0.16	-0.11	-0.14	-0.13	-0.20	-0.07	-0.11	-0.15	-0.11	-0.16	-0.11	-0.15	-0.12	-0.10	-0.13	-0.08
**Sample size (n):**								15															15							

Statistics Analysis

Test: unpaired *t*-test

% of contraction force	Normal		Affected	
	Average	SD	Average	SD
0-10	-0.01	0.01	0.00	0.00
10-20	-0.49	0.22	-0.33	0.10
20-30	-1.04	0.22	-0.68	0.16
30-40	-1.43	0.29	-1.00	0.23
40-50	-1.83	0.37	-1.29	0.31
50-60	-2.16	0.41	-1.52	0.38
60-70	-2.29	0.42	-1.62	0.42
70-80	-2.11	0.39	-1.55	0.41
80-90	-1.52	0.26	-1.23	0.34
90-100	-0.18	0.03	-0.12	0.03

% of contraction force	P Value
0-10	0.0342
10-20	0.0342
20-30	0.0002
30-40	0.0008
40-50	0.0011
50-60	0.0009
60-70	0.0010
70-80	0.0027
80-90	0.0342
90-100	0.0002

Disease Models & Mechanisms • Supplementary information

Fig. 5F	
FWHM at contraction (msec)	
Normal	Affected
150.50	170.80
127.70	142.70
141.90	104.40
260.30	100.70
231.30	152.90
155.00	100.00
135.80	121.00
191.40	108.10
174.20	128.30
165.90	109.00
168.10	135.80
153.70	132.60
174.10	151.50
155.00	141.10
175.10	141.00

**Average:**	170.70	129.30
**SD:**	35.14	21.56
**Sample size (n):**	15	15

Statistics Analysis

Test: unpaired *t* -test

P value: 0.0006

Fig. 5H	
FWHM at relaxation (msec)	
Normal	Affected
49.00	45.70
55.90	35.30
46.70	38.20
40.70	36.30
44.10	48.60
57.00	34.30
45.60	46.30
55.60	40.10
47.00	41.30
52.40	45.30
88.50	47.30
45.50	45.60
40.80	51.50
41.10	41.00
46.40	48.00

**Average:**	50.42	42.99
**SD:**	11.83	5.32
**Sample size (n):**	15	15

Statistics Analysis

Test: unpaired *t* -test

P value: 0.0347

Fig. 6A

Muscle force (N)

Stimulation frequency (Hz)	Normal														
	Dog # 1	Dog # 2	Dog # 3	Dog # 4	Dog # 5	Dog # 6	Dog # 7	Dog # 8	Dog # 9	Dog # 10	Dog # 11	Dog # 12	Dog # 13	Dog # 14	Dog # 15
5	25.88	20.71	13.74	18.52	16.55	17.12	15.66	14.62	18.60	22.33	12.31	16.00	18.31	18.81	14.43
20	102.23	125.68	103.80	77.83	88.57	93.31	74.31	73.87	90.07	103.33	63.98	92.45	88.29	93.01	59.98
40	150.09	146.44	115.42	111.69	125.25	133.69	105.33	107.39	139.54	163.42	99.66	141.89	134.86	146.52	93.69
60	169.09	154.48	118.22	125.97	141.04	146.29	114.31	119.98	153.84	184.54	109.69	157.06	148.60	164.65	108.86
80	175.11	158.54	119.83	130.69	140.78	151.76	118.85	124.81	157.80	191.39	112.81	162.37	152.97	170.67	114.53
100	175.07	160.13	120.93	132.15	140.32	152.40	123.33	123.38	156.63	193.38	113.02	163.49	152.18	172.56	117.35
120	166.62	158.60	120.43	129.53	140.06	152.91	127.66	118.95	149.78	193.25	110.35	162.13	145.97	173.49	117.35

Stimulation frequency (Hz)	Affected													
	Dog # 1	Dog # 2	Dog # 3	Dog # 4	Dog # 5	Dog # 6	Dog # 7	Dog # 8	Dog # 9	Dog # 10	Dog # 11	Dog # 12	Dog # 13	Dog # 14
5	6.33	13.49	13.75	6.13	12.21	11.50	9.14	15.02	10.56	10.67	11.46	11.76	12.03	10.18
20	27.59	59.19	55.04	26.82	50.10	66.33	28.40	64.55	52.17	66.71	39.16	53.04	52.44	51.05
40	59.00	89.34	100.61	45.79	74.00	101.18	56.83	94.79	102.54	87.18	65.74	102.85	81.48	87.03
60	72.09	101.28	119.55	55.17	83.27	114.58	67.12	108.20	122.60	94.85	75.81	120.25	89.32	103.72
80	76.15	103.00	125.77	56.23	85.63	119.73	71.10	112.45	129.57	97.75	79.70	124.30	93.53	110.46
100	77.37	98.95	124.93	54.03	82.59	121.43	71.80	110.40	130.76	97.63	80.62	122.30	93.76	111.13
120	76.84	92.03	122.00	50.51	76.75	121.56	71.92	105.50	130.30	97.99	80.19	116.13	92.60	109.50

Sample size (n): Normal 15; Affected 14

Statistics Analysis

Test: unpaired *t*-test

% of contraction force	Normal		Affected	
	Average	SD	Average	SD
5	17.57	3.52	11.02	2.53
20	88.71	16.97	49.47	13.89
40	127.66	21.01	82.03	18.90
60	141.11	23.67	94.84	21.66
80	145.53	24.47	98.96	22.81
100	146.42	24.32	98.41	22.98
120	144.47	23.87	95.99	22.96

% of contracti on force	P Value
0-10	0.0000
10-20	0.0000
20-30	0.0000
30-40	0.0000
40-50	0.0000
50-60	0.0000
60-70	0.0000

Fig. 6B

Muscle force (N/cm^2)

Stimulation frequency (Hz)	Normal														
	Dog # 1	Dog # 2	Dog # 3	Dog # 4	Dog # 5	Dog # 6	Dog # 7	Dog # 8	Dog # 9	Dog # 10	Dog # 11	Dog # 12	Dog # 13	Dog # 14	Dog # 15
5	1.53	1.59	1.23	1.45	1.24	1.35	1.26	1.21	1.30	1.31	1.08	0.95	1.11	1.16	1.43
20	6.06	9.63	9.29	6.10	6.65	7.37	5.99	6.14	6.32	6.05	5.61	5.49	5.34	5.75	5.94
40	8.90	11.22	10.33	8.75	9.41	10.56	8.49	8.92	9.78	9.57	8.74	8.43	8.16	9.06	9.27
60	10.03	11.83	10.58	9.87	10.60	11.56	9.21	9.97	10.79	10.81	9.62	9.33	9.00	10.19	10.77
80	10.38	12.14	10.72	10.24	10.58	11.99	9.58	10.37	11.06	11.21	9.90	9.64	9.26	10.56	11.34
100	10.38	12.27	10.82	10.35	10.54	12.04	9.94	10.25	10.98	11.33	9.92	9.71	9.21	10.68	11.61
120	9.88	12.15	10.77	10.15	10.52	12.08	10.29	9.88	10.50	11.32	9.68	9.63	8.84	10.73	11.62

Stimulation frequency (Hz)	Affected													
	Dog # 1	Dog # 2	Dog # 3	Dog # 4	Dog # 5	Dog # 6	Dog # 7	Dog # 8	Dog # 9	Dog # 10	Dog # 11	Dog # 12	Dog # 13	Dog # 14
5	0.75	1.15	1.12	0.78	1.55	0.85	0.97	1.41	0.97	0.82	0.91	0.79	1.36	1.02
20	3.26	5.06	4.47	3.41	6.36	4.93	3.01	6.08	4.78	5.15	3.12	3.56	5.92	5.12
40	6.98	7.63	8.17	5.83	9.39	7.52	6.03	8.93	9.39	6.73	5.24	6.91	9.20	8.73
60	8.53	8.65	9.71	7.02	10.56	8.51	7.12	10.19	11.23	7.32	6.05	8.07	10.08	10.40
80	9.01	8.80	10.22	7.15	10.86	8.89	7.54	10.59	11.87	7.54	6.36	8.35	10.56	11.08
100	9.15	8.46	10.15	6.88	10.48	9.02	7.61	10.40	11.97	7.53	6.43	8.21	10.58	11.15
120	9.09	7.86	9.91	6.43	9.74	9.03	7.63	9.93	11.93	7.56	6.40	7.80	10.45	10.98

Sample size (n): Normal 15; Affected 14

Statistics Analysis

Test: unpaired *t*-test

% of contraction force	Normal		Affected	
	Average	SD	Average	SD
5	1.28	0.17	1.03	0.25
20	6.52	1.29	4.59	1.14
40	9.31	0.86	7.62	1.39
60	10.28	0.82	8.82	1.58
80	10.60	0.84	9.20	1.69
100	10.67	0.86	9.14	1.69
120	10.54	0.94	8.91	1.68

% of contracti on force	P Value
0-10	0.0055
10-20	0.0016
20-30	0.0017
30-40	0.0055
40-50	0.0084
50-60	0.0055
60-70	0.0055

Fig. 6C

% of Maximum force

Stimulation frequency (Hz)	Normal														
	Dog # 1	Dog # 2	Dog # 3	Dog # 4	Dog # 5	Dog # 6	Dog # 7	Dog # 8	Dog # 9	Dog # 10	Dog # 11	Dog # 12	Dog # 13	Dog # 14	Dog # 15
5	14.78	12.93	11.36	14.01	11.73	11.20	12.27	11.72	11.79	11.55	10.89	9.79	11.97	10.84	12.30
20	58.38	78.48	85.83	58.89	62.80	61.02	58.21	59.18	57.08	53.43	56.61	56.55	57.71	53.61	51.11
40	85.71	91.45	95.44	84.52	88.80	87.43	82.51	86.04	88.43	84.51	88.18	86.79	88.16	84.46	79.83
60	96.57	96.47	97.77	95.32	100.00	95.67	89.55	96.13	97.49	95.43	97.06	96.07	97.14	94.91	92.76
80	100.00	99.00	99.09	98.89	99.82	99.25	93.10	100.00	100.00	98.97	99.81	99.32	100.00	98.38	97.59
100	99.98	100.00	100.00	100.00	99.49	99.66	96.61	98.86	99.26	100.00	100.00	100.00	99.48	99.47	100.00
120	95.15	99.04	99.59	98.01	99.31	100.00	100.00	95.31	94.92	99.93	97.64	99.17	95.42	100.00	100.00

Stimulation frequency (Hz)	Affected													
	Dog # 1	Dog # 2	Dog # 3	Dog # 4	Dog # 5	Dog # 6	Dog # 7	Dog # 8	Dog # 9	Dog # 10	Dog # 11	Dog # 12	Dog # 13	Dog # 14
5	8.18	13.09	10.94	10.91	14.26	9.46	12.71	13.35	8.08	10.89	14.22	9.46	12.83	9.16
20	35.66	57.47	43.76	47.71	58.51	54.57	39.49	57.40	39.90	68.08	48.58	42.67	55.93	45.94
40	76.26	86.74	79.99	81.44	86.42	83.24	79.01	84.30	78.41	88.97	81.54	82.74	86.91	78.31
60	93.18	98.33	95.06	98.13	97.25	94.26	93.33	96.22	93.76	96.79	94.04	96.74	95.27	93.33
80	98.42	100.00	100.00	100.00	100.00	98.50	98.86	100.00	99.09	99.75	98.87	100.00	99.76	99.39
100	100.00	96.07	99.34	96.09	96.46	99.90	99.84	98.18	100.00	99.63	100.00	98.38	100.00	100.00
120	99.31	89.35	97.00	89.84	89.63	100.00	100.00	93.81	99.65	100.00	99.47	93.42	98.77	98.53

Sample size (n): Normal 15; Affected 14

Statistics Analysis

Test: unpaired *t*-test

% of contraction force	Normal		Affected	
	Average	SD	Average	SD
5	11.94	1.24	11.25	2.16
20	60.59	9.34	49.69	9.19
40	86.82	3.70	82.45	3.85
60	95.89	2.37	95.41	1.83
80	98.88	1.74	99.47	0.61
100	99.52	0.88	98.85	1.55
120	98.23	2.02	96.34	4.22

% of contracti on force	P Value
0-10	0.3515
10-20	0.0152
20-30	0.0152
30-40	0.5510
40-50	0.3369
50-60	0.2818
60-70	0.2818

Fig. 6D

Muscle force (% of initial)

ECC cycle	Normal														
	Dog # 1	Dog # 2	Dog # 3	Dog # 4	Dog # 5	Dog # 6	Dog # 7	Dog # 8	Dog # 9	Dog # 10	Dog # 11	Dog # 12	Dog # 13	Dog # 14	Dog # 15
1	100.00	100.00	100.00	100.00	100.00	100.00	100.00	100.00	100.00	100.00	100.00	100.00	100.00	100.00	100.00
2	100.51	87.39	99.36	100.12	104.18	103.42		98.55	99.27	95.47		98.92	98.33	101.29	
3	98.90	86.56	98.73	97.61	102.99	89.75		97.40	98.11	94.75		98.32	97.41	74.63	
4	99.24	86.63	98.07	96.93	101.24	78.34		96.40	97.54	94.22		98.24	97.34	83.10	
5	101.84	87.20	97.47	95.28	96.39	89.23		96.60	96.90	93.43		98.08	97.30	81.04	
6	99.87	86.00	96.24	92.99	94.88	89.92		96.86	96.50	92.83		98.14	96.80	80.21	
7	98.30	91.68	95.66	92.45	95.71	90.95		96.65	96.01	92.33		98.48	96.64	81.13	
8	100.31	90.72	94.23	92.00	97.40	91.19		96.19	95.53	91.09		98.46	96.70	79.78	
9	99.35	89.03	94.58	90.19	96.72	91.02		96.03	95.09	90.57		98.41	96.72	77.95	
10	97.81	87.36	94.32	87.67	96.06	88.77		96.34	94.76	89.34		97.72	96.25	76.81	

ECC cycle	Affected														
	Dog # 1	Dog # 2	Dog # 3	Dog # 4	Dog # 5	Dog # 6	Dog # 7	Dog # 8	Dog # 9	Dog # 10	Dog # 11	Dog # 12	Dog # 13	Dog # 14	Dog # 15
1	100.00	100.00	100.00	100.00	100.00	100.00	100.00	100.00	100.00	100.00	100.00	100.00	100.00	100.00	100
2	87.18	89.94	78.73	87.72	89.82	87.32	93.14	82.97	88.63	94.26	97.10	92.25	93.23	97.08	87.67
3	78.94	81.95	53.60	76.43	81.32	77.90	80.60	67.71	79.39	89.84	84.88	84.18	90.21	92.57	77.46
4	73.42	77.24	46.28	67.93	74.78	71.38	67.06	54.16	71.59	85.94	64.84	74.48	87.73	87.25	70.43
5	69.04	70.85	39.76	60.84	69.52	67.45	56.78	43.61	65.39	82.46	54.38	66.80	83.65	83.32	65.32
6	66.18	67.54	35.39	55.97	64.66	63.42	50.15	34.77	59.85	78.54	48.58	60.83	80.98	79.89	61.57
7	63.15	66.96	34.31	52.23	60.89	60.68	45.87	28.74	55.60	73.71	44.26	55.69	78.48	76.47	58.42
8	60.22	64.98	31.45	48.95	57.84	58.03	42.23	1.13	51.94	69.92	41.24	51.98	75.48	75.92	55.44
9	58.36	59.20	29.18	46.45	55.49	55.37	39.04	0.29	48.89	64.61	39.84	48.14	72.25	75.35	52.86
10	57.04	56.41	28.07	44.02	52.87	53.62	37.14	0.04	46.59	62.49	38.75	46.28	69.39	72.51	51.09

Sample size (n): Normal 15; Affected 15

Statistics Analysis

Test: unpaired *t*-test

ECC cycle	Normal		Affected	
	Average	SD	Average	SD
1	100.00	0.00	100.00	0.00
2	98.90	4.30	89.80	4.98
3	94.60	7.65	79.80	9.56
4	93.94	7.20	71.63	11.31
5	94.23	5.72	65.28	12.98
6	93.44	5.63	60.55	14.12
7	93.83	4.74	57.03	14.43
8	93.63	5.37	52.45	18.88
9	92.97	5.83	49.69	18.35
10	91.93	6.19	47.75	17.74

ECC cycle	P Value
1	
2	0.0000
3	0.0002
4	0.0000
5	0.0000
6	0.0000
7	0.0000
8	0.0000
9	0.0000
10	0.0000

Disease Models & Mechanisms • Supplementary information

Fig. 7B

MyHC isoforms	% of myofibers													
	Normal						Affected							
	Dog # 1	Dog # 2	Dog # 3	Dog # 4	Dog # 5	Dog # 6	Dog # 1	Dog # 2	Dog # 3	Dog # 4	Dog # 5	Dog # 6	Dog # 7	Dog # 8
I	71.68	65.74	76.16	61.86	68.30	57.81	28.37	34.90	37.87	25.92	27.95	24.94	23.71	15.83
I/IIa	0.48	0.00	1.02	0.00	0.94	0.48	19.61	12.89	6.84	14.95	20.07	2.68	2.85	14.29
IIa	27.84	34.26	22.82	38.14	30.76	41.71	52.02	52.21	55.28	59.13	51.98	72.38	73.44	69.88
**Sample size (n):**	6						8							

MyHC isoforms	Normal		Affected	
	Average	SD	Average	SD
I	66.93	6.63	27.44	6.79
I/IIa	0.49	0.44	11.77	6.91
IIa	32.59	6.90	60.79	9.55

Statistics Analysis

Test: unpaired *t* -test

MyHC isoforms	P Value
I	0.0000
I/IIa	0.0019
IIa	0.0001

Fig. 7C

MyHC isoforms	Relative percentage (%)					
	Normal			Affected		
	Dog # 1	Dog # 2	Dog # 3	Dog # 1	Dog # 2	Dog # 3
I	55.55	55.54	47.69	28.60	33.97	44.62
IIa	44.45	44.46	52.31	71.40	66.03	55.38
**Sample size (n):**	3			3		

MyHC isoforms	Normal		Affected	
	Average	SD	Average	SD
I	52.93	4.54	35.73	8.15
IIa	47.07	4.54	64.27	8.15

Statistics Analysis

Test: unpaired *t* -test

MyHC isoforms	P Value
I	0.0331
IIa	0.0331

Fig. 7B

Transcript	Transcript copy (copy/ng)												
	Normal						Affected						
	Dog # 1	Dog # 2	Dog # 3	Dog # 4	Dog # 5	Dog # 6	Dog # 1	Dog # 2	Dog # 3	Dog # 4	Dog # 5	Dog # 6	Dog # 7
MyHC7	1.4E+05	7.5E+04	1.3E+05	5.6E+04	1.1E+05	8.3E+04	4.8E+04	4.0E+04	2.9E+04	3.4E+04	7.7E+04	5.0E+04	9.0E+04
MyHC2	9.0E+04	5.9E+04	1.0E+05	5.4E+04	4.6E+04	4.6E+04	1.1E+05	6.4E+04	2.7E+04	7.1E+04	1.2E+05	1.5E+05	1.3E+05
MyHC1	8.4E+02	5.2E+02	1.3E+03	4.3E+02	4.8E+02	3.5E+02	4.6E+02	4.6E+03	9.9E+02	3.1E+03	2.0E+03		1.4E+03
MyHC4	1.0E+00	1.0E+00	0.0E+00	0.0E+00	0.0E+00	0.0E+00	2.0E+01	2.3E+01	1.8E+01	9.0E+00	4.0E+00	1.6E+01	3.1E+01
**Sample size (n):**	6						8						

Transcript	Normal		Affected	
	Average	SD	Average	SD
MyHC7	1.0E+05	3.5E+04	5.2E+04	2.3E+04
MyHC2	6.7E+04	2.4E+04	9.5E+04	4.2E+04
MyHC1	6.6E+02	3.7E+02	2.1E+03	1.5E+03
MyHC4	3.3E-01	5.2E-01	1.7E+01	8.9E+00

Statistics Analysis

Test: unpaired *t* -test

Transcript	P Value
MyHC7	0.0208
MyHC2	0.2547
MyHC1	0.0177
MyHC4	0.0021

Disease Models & Mechanisms • Supplementary information

Table 1

Dog name	Age (Year)	Body weigh (kg)	ECU muscle weight (g)	ECU weight/body weight (g/kg)	ECU muscle lenght (cm)	pCSA (cm^2)
Normal	2.16	34.47	12.93	0.38	15.96	16.86
	2.28	19.50	9.22	0.47	14.70	13.06
	2.50	17.24	6.09	0.35	11.34	11.18
	2.62	18.51	8.50	0.46	13.86	12.77
	2.82	20.41	9.40	0.46	14.70	13.31
	2.82	20.41	8.94	0.44	14.70	12.66
	2.82	15.38	7.26	0.47	12.18	12.41
	3.07	16.92	6.80	0.40	11.76	12.04
	3.09	22.72	10.36	0.46	15.12	14.26
	3.19	23.59	12.40	0.53	15.12	17.07
	3.20	21.45	7.13	0.33	13.02	11.40
	3.40	20.41	12.23	0.60	15.12	16.84
	3.42	20.46	12.00	0.59	15.12	16.52
	3.47	27.22	12.72	0.47	16.38	16.16
	3.54	19.73	6.32	0.32	13.02	10.10
**Min**	2.16	15.38	6.09	0.32	11.34	10.10
**Max**	3.54	34.47	12.93	0.60	16.38	17.07
**Average**	2.96	21.23	9.49	0.45	14.14	13.78
**SD**	0.43	4.65	2.48	0.08	1.54	2.35
Affected	2.48	15.24	5.63	0.37	13.86	8.46
	2.55	21.59	7.32	0.34	13.02	11.70
	2.75	20.09	8.13	0.40	14.28	11.85
	2.78	20.09	9.44	0.47	15.96	12.31
	2.86	15.83	4.44	0.28	11.76	7.86
	2.90	15.20	5.09	0.33	13.44	7.88
	3.02	19.87	9.78	0.49	15.12	13.46
	3.02	14.88	6.09	0.41	13.44	9.43
	3.22	20.41	7.50	0.37	14.70	10.62
	3.47	22.90	8.37	0.37	15.96	10.92
	3.48	22.13	8.63	0.39	13.86	12.96
	3.49	17.41	7.59	0.44	12.60	12.54
	3.50	29.84	11.72	0.39	16.38	14.89
	3.65	14.74	5.72	0.39	13.44	8.86
	3.73	19.00	6.84	0.36	14.28	9.97
**Min**	2.78	14.74	4.44	0.28	11.76	7.86
**Max**	3.65	29.84	11.72	0.49	16.38	14.89
**Average**	3.13	19.28	7.49	0.39	14.14	10.91
**SD**	0.41	4.05	1.95	0.05	1.31	2.15
**P value**	0.2913	0.2318	0.0206	0.0225	1.0000	0.0016

Disease Models & Mechanisms • Supplementary information

Fig. S1

Dog name	Body weigh (kg)	ECU muscle weight (g)	pCSA (cm^2)
Normal	34.47	12.93	16.86
	19.50	9.22	13.06
	17.24	6.09	11.18
	18.51	8.50	12.77
	20.41	9.40	13.31
	20.41	8.94	12.66
	15.38	7.26	12.41
	16.92	6.80	12.04
	22.72	10.36	14.26
	23.59	12.40	17.07
	21.45	7.13	11.40
	20.41	12.23	16.84
	20.46	12.00	16.52
	27.22	12.72	16.16
	19.73	6.32	10.10
Affected	15.24	5.63	8.46
	21.59	7.32	11.70
	20.09	8.13	11.85
	20.09	9.44	12.31
	15.83	4.44	7.86
	15.20	5.09	7.88
	19.87	9.78	13.46
	14.88	6.09	9.43
	20.41	7.50	10.62
	22.90	8.37	10.92
	22.13	8.63	12.96
	17.41	7.59	12.54
	29.84	11.72	14.89
	14.74	5.72	8.86
	19.00	6.84	9.97

Fig. S3	
Pt/Po	
Normal	Affected
0.12	0.07
0.10	0.12
0.12	0.09
0.10	0.10
0.09	0.11
0.09	0.08
0.10	0.10
0.11	0.12
0.11	0.07
0.11	0.11
0.08	0.08
0.10	0.12
0.09	
0.11	

	Normal	Affected
**Average:**	0.10	0.10
**SD:**	0.01	0.02
**Sample size (n):**	14	12

Statistics Analysis

Test: unpaired *t* -test

P value: 0.4575

Fig. S6A	
Pt/TPT	
Normal	Affected
0.21	0.10
0.16	0.21
0.21	0.21
0.19	0.09
0.17	0.14
0.13	0.16
0.18	0.16
0.24	0.21
0.30	0.15
0.19	0.18
0.19	0.17
0.18	0.20
0.18	
0.21	

	Normal	Affected
**Average:**	0.20	0.17
**SD:**	0.04	0.04
**Sample size (n):**	14	12

Statistics Analysis

Test: unpaired *t* -test

P value: 0.0470

Fig. S6B	
Pt/1/2RT	
Normal	Affected
0.29	0.14
0.20	0.26
0.21	0.23
0.24	0.09
0.19	0.16
0.15	0.18
0.23	0.21
0.29	0.29
0.42	0.17
0.24	0.21
0.26	0.22
0.22	0.19
0.17	
0.25	

	Normal	Affected
**Average:**	0.24	0.20
**SD:**	0.07	0.05
**Sample size (n):**	14	12

Statistics Analysis

Test: unpaired *t* -test

P value: 0.0785

Fig. S6C	
Po/TPT	
Normal	Affected
0.20	0.10
0.15	0.17
0.17	0.15
0.19	0.21
0.16	0.09
0.16	0.17
0.24	0.15
0.15	0.11
0.20	0.14
0.19	0.16
0.18	0.12
0.14	0.11
	0.15
	0.13
	0.14

	Normal	Affected
**Average:**	0.18	0.14
**SD:**	0.03	0.03
**Sample size (n):**	12	15

Statistics Analysis

Test: unpaired *t* -test

P value: 0.0036

Fig. S6D	
Po/1/2RT	
Normal	Affected
1.49	0.81
1.35	1.06
1.18	1.00
1.27	1.48
1.34	0.56
1.39	0.82
1.05	1.14
1.26	0.81
1.43	1.14
1.83	1.43
1.20	1.07
1.40	0.95
1.23	1.31
1.58	1.01
1.17	1.10

	Normal	Affected
**Average:**	1.35	1.05
**SD:**	0.19	0.24
**Sample size (n):**	15	15

Statistics Analysis

Test: unpaired *t* -test

P value: 0.0009

Disease Models & Mechanisms • Supplementary information

Table S1

Dog name	Optimal resting tension (g)	Optimal stimulation current (mA)	Optimal Stimulation duration (msec)	Optimal stimulation frequency (Hz)
Normal	1100	20	800	80
	1100	60	800	80
	1300	600	500	80
	1500	40	800	100
	1600	40	800	100
	1400	50	500	100
	900	60	800	100
	1463	60	800	100
	700	80	800	100
	800	100	800	100
	1000	200	800	100
	1000	40	800	120
	1500	40	800	120
	2000	40	800	120
	1500	80	800	120
**Min**	700.00	20.00	500.00	80.00
**Max**	2000.00	600.00	800.00	120.00
**Average**	1257.53	100.67	760.00	101.33
**SD**	352.49	144.54	105.56	14.07
Affected	600	60	500	80
	1000	20	600	80
	600	60	600	80
	600	80	600	80
	600	100	600	80
	1600	20	800	80
	400g	40	500	100
	1100	20	800	100
	700	20	800	100
	300	40	800	100
	500	80	800	100
	500	200	800	100
	500	300	800	100
	900	40	800	120
	700	80	800	120
**Min**	300.00	20.00	500.00	80.00
**Max**	1600.00	300.00	800.00	120.00
**Average**	728.57	77.33	706.67	94.67
**SD**	329.17	77.04	122.28	14.07
**P value**	0.0003	0.5855	0.2115	0.2052

Disease Models & Mechanisms • Supplementary information

Table S4

Dog name	Age (Year)	Body weigh (kg)	ECU muscle weight (g)	ECU weight/body weight (g/kg)	ECU muscle lenght (cm)	pCSA (cm^2)	Po (N)	Po/ECU weight (N/g)	Po/pCSA (N/cm^2)
	2.16	34.47	12.93	0.38	15.96	16.86	175.11	13.54	10.38
	2.28	19.50	9.22	0.47	14.70	13.06	160.13	17.37	12.27
	2.50	17.24	6.09	0.35	11.34	11.18	120.93	19.86	10.82
	2.62	18.51	8.50	0.46	13.86	12.77	132.15	15.55	10.35
	2.82	20.41	9.40	0.46	14.70	13.31	141.04	15.00	10.60
	2.82	20.41	8.94	0.44	14.70	12.66	152.91	17.10	12.08
	2.82	15.38	7.26	0.47	12.18	12.41	128.56	17.71	10.36
**Normal**	3.07	16.92	6.80	0.40	11.76	12.04	123.38	18.14	10.25
	3.09	22.72	10.36	0.46	15.12	14.26	157.80	15.23	11.06
	3.19	23.59	12.40	0.53	15.12	17.07	193.38	15.60	11.33
	3.20	21.45	7.13	0.33	13.02	11.40	113.02	15.85	9.92
	3.40	20.41	12.23	0.60	15.12	16.84	163.49	13.37	9.71
	3.42	20.46	12.00	0.59	15.12	16.52	152.97	12.75	9.26
	3.47	27.22	12.72	0.47	16.38	16.16	173.49	13.64	10.73
	3.54	19.73	6.32	0.32	13.02	10.10	117.35	18.57	11.62
**Min**	2.16	15.38	6.09	0.32	11.34	10.10	113.02	12.75	9.26
**Max**	3.54	34.47	12.93	0.60	16.38	17.07	193.38	19.86	12.27
**Average**	2.96	21.23	9.49	0.45	14.14	13.78	147.05	15.95	10.72
**SD**	0.43	4.65	2.48	0.08	1.54	2.35	24.11	2.12	0.84
	2.48	15.24	5.63	0.37	13.86	8.46	77.37	13.74	9.15
	2.55	21.59	7.32	0.34	13.02	11.70	103.00	14.07	8.80
	2.75	20.09	8.13	0.40	14.28	11.85	72.02	8.86	6.08
	2.78	20.09	9.44	0.47	15.96	12.31	125.77	13.32	10.22
	2.86	15.83	4.44	0.28	11.76	7.86	56.23	12.66	7.15
	2.90	15.20	5.09	0.33	13.44	7.88	85.63	16.82	10.86
	3.02	19.87	9.78	0.49	15.12	13.46	121.56	12.43	9.03
**Affected**	3.02	14.88	6.09	0.41	13.44	9.43	71.92	11.81	7.63
	3.22	20.41	7.50	0.37	14.70	10.62	112.45	14.99	10.59
	3.47	22.90	8.37	0.37	15.96	10.92	130.71	15.62	11.97
	3.48	22.13	8.63	0.39	13.86	12.96	97.99	11.35	7.56
	3.49	17.41	7.59	0.44	12.60	12.54	80.62	10.62	6.43
	3.50	29.84	11.72	0.39	16.38	14.89	124.30	10.61	8.35
	3.65	14.74	5.72	0.39	13.44	8.86	93.76	16.39	10.58
	3.73	19.00	6.84	0.36	14.28	9.97	111.13	16.25	11.15
**Min**	2.78	14.74	4.44	0.28	11.76	7.86	56.23	8.86	6.08
**Max**	3.65	29.84	11.72	0.49	16.38	14.89	130.71	16.82	11.97
**Average**	3.13	19.28	7.49	0.39	14.14	10.91	97.63	13.30	9.04
**SD**	0.41	4.05	1.95	0.05	1.31	2.15	23.13	2.41	1.82
**P value**	0.2913	0.2318	0.0206	0.0225	1.0000	0.0016	0.0000	0.0034	0.0031

Table S5

**Dog name**	**% of Length stretch**	**Stretch rate**
**Normal**	5.00	7.98
	5.00	7.35
	5.00	5.67
	5.00	6.93
	5.00	7.35
	5.00	7.35
	5.00	6.09
	5.00	5.88
	5.00	7.56
	5.00	7.56
	5.00	6.51
	5.00	7.56
	5.00	7.56
	5.00	8.19
	5.00	6.51
**Min**	5.00	5.67
**Max**	5.00	8.19
**Average**	5.00	7.07
**SD**	0.00	0.77
**Affected**	5.00	6.93
	5.00	6.51
	5.00	7.14
	5.00	7.98
	5.00	5.88
	5.00	6.72
	5.00	7.56
	5.00	6.72
	5.00	7.35
	5.00	7.98
	5.00	6.93
	5.00	6.30
	5.00	8.19
	5.00	6.72
	5.00	7.14
**Min**	5.00	5.88
**Max**	5.00	8.19
**Average**	5.00	7.07
**SD**	0.00	0.65
**P value**		1.00

Disease Models & Mechanisms • Supplementary information
